# Germinal Center Kinases SmKIN3 and SmKIN24 Are Associated with the *Sordaria macrospora* Striatin-Interacting Phosphatase and Kinase (STRIPAK) Complex

**DOI:** 10.1371/journal.pone.0139163

**Published:** 2015-09-29

**Authors:** Stefan Frey, Eva J. Reschka, Stefanie Pöggeler

**Affiliations:** 1 Institute of Microbiology and Genetics, Department of Genetics of Eukaryotic Microorganisms, Georg-August-University Göttingen, Göttingen, Germany; 2 Göttingen Center for Molecular Biosciences (GZMB), Georg-August-University Göttingen, Göttingen, Germany; Friedrich Schiller University, GERMANY

## Abstract

The striatin-interacting phosphatase and kinase (STRIPAK) complex is composed of striatin, protein phosphatase PP2A and protein kinases that regulate development in animals and fungi. In the filamentous ascomycete *Sordaria macrospora*, it is required for fruiting-body development and cell fusion. Here, we report on the presence and function of STRIPAK-associated kinases in ascomycetes. Using the mammalian germinal center kinases (GCKs) MST4, STK24, STK25 and MINK1 as query, we identified the two putative homologs SmKIN3 and SmKIN24 in *S*. *macrospora*. A BLASTP search revealed that both kinases are conserved among filamentous ascomycetes. The physical interaction of the striatin homolog PRO11 with SmKIN3 and SmKIN24 were verified by yeast two-hybrid (Y2H) interaction studies and for SmKIN3 by co-Immunoprecipitation (co-IP). *In vivo* localization found that both kinases were present at the septa and deletion of both *Smkin3* and *Smkin24* led to abnormal septum distribution. While deletion of *Smkin3* caused larger distances between adjacent septa and increased aerial hyphae, deletion of *Smkin24* led to closer spacing of septa and to sterility. Although phenotypically distinct, both kinases appear to function independently because the double-knockout strain ΔSmkin3/ΔSmkin24 displayed the combined phenotypes of each single-deletion strain.

## Introduction

In filamentous fungi, the development of multicellular fruiting bodies requires highly conserved differentiation processes and is essential for sexual reproduction [1]. The filamentous ascomycete *Sordaria macrospora* is a well-established model organism for studying this process [2, 3]. *S*. *macrospora* is a haplont with a homothallic life style and does not require a mating partner for sexual reproduction [4]. In contrast to many other filamentous ascomycetes, *S*. *macrospora* produces no asexual spores [5]. The life cycle begins with the germination of ascospores that develop into a vegetative, two-dimensional mycelium that forms ascogonia after three days representing the female gametangia. These hyphal coils develop into pre-fruiting bodies named protoperithecia within 5 days. After self-fertilization followed by karyogamy, meiosis and a postmeiotic mitosis by day seven, pre-fruiting bodies become mature fruiting bodies (perithecia) that harbor asci containing eight aligned ascospores per ascus. Ascospores are then released from the pear-shaped fruiting body by increased turgor pressure of the asci [6, 7]. A genetic screen of *S*. *macrospora* identified several “pro” mutants characterized by a developmental arrest between protoperithecia and perithecia formation [3]. Complementation of these mutants and whole genome sequencing identified several PRO proteins involved in fruiting-body development [5, 8–13].

**Fig 1:**
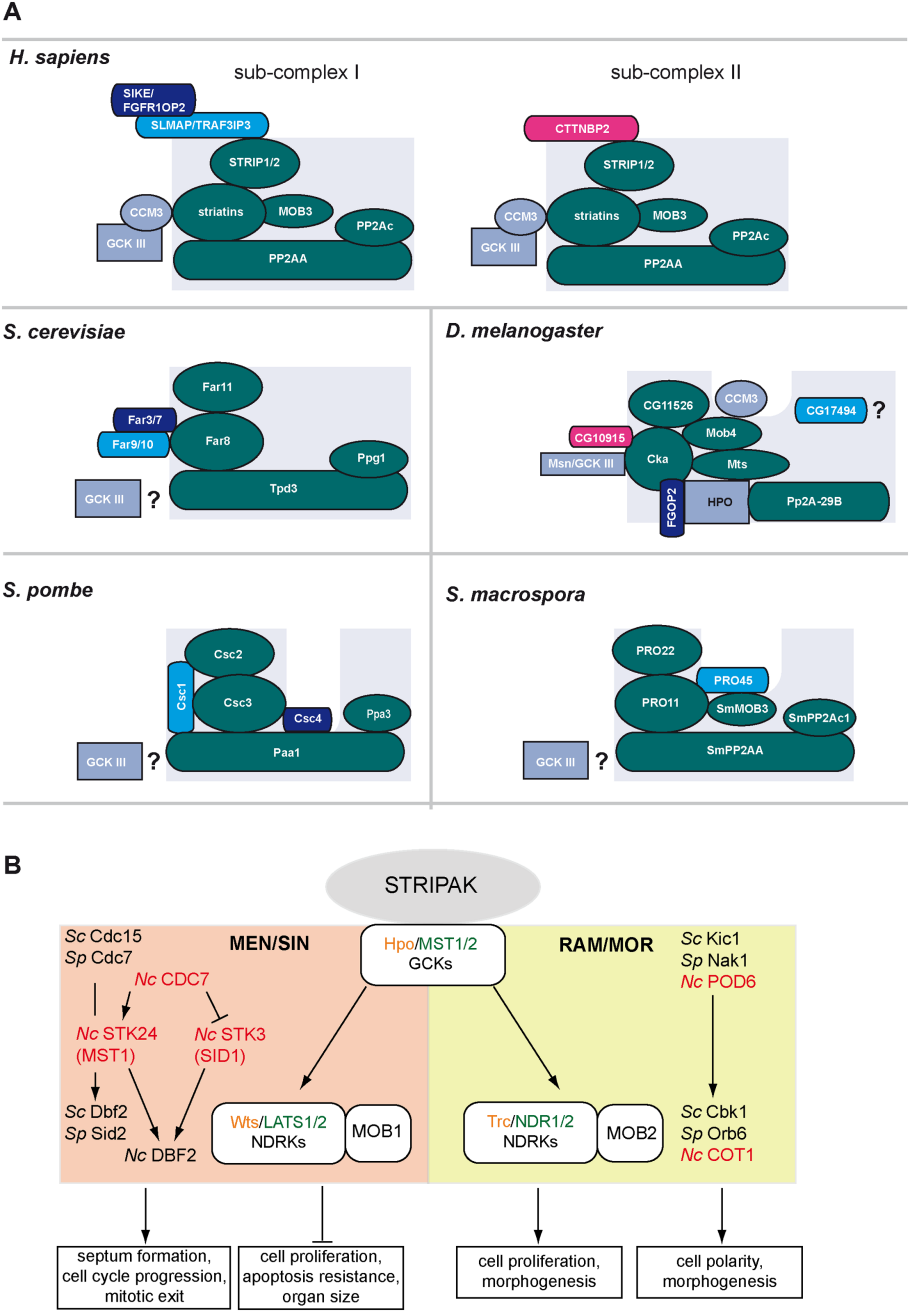
Schematic representation of mammalian, *D*. *melanogaster* and fungal STRIPAK complex and STRIPAK-like complexes (A) and structure of the Hippo network in animals and the MEN/SIN as well as the RAM/MOR signaling pathway in fungi (B). (A) Core components of the STRIPAK and STRIPAK-like complex (striatin, Mob3, catalytic and scaffold subunit of the phosphatase PP2A, and STRIP proteins and the respective homologs) are depicted in green, putative homologs of the cortactin-binding protein CTTNBP2 are shown in red, putative homologs of SLMAP in light blue and putative SIKE/FGFR1OP2 in dark blue. STRIPAK GCKs and the associated CCM3 protein are indicated in violet. The arrangements of the subunits in the respective are deduced from [15, 20, 27–34]. (B) Components of the Hippo pathway in *D*. *melanogaster* and mammals are indicated in orange and green, respectively. Homologous components of the *S*. *cerevisiae* (*Sc*) MEN and RAM network and the corresponding *S*. *pombe* (*Sp*) SIN and MOR network are shown in the left and right boxes. Components from the filamentous fungus *N*. *crassa* (*Nc*) are shown in red according to [35]. The figure was modified from [23].

One of these *pro* genes, namely *pro11*, encodes a homolog of the mammalian striatin protein family [8]. Mammalian striatins are multidomain proteins possessing a caveolin-binding domain, a coiled-coil domain, a calmodulin binding domain at the N-terminus and a C-terminal WD40 repeat region. They were initially identified as a novel group of phosphatase PP2A regulatory B”‘ subunits with roles in both signaling and trafficking [14]. Moreover, striatins serve as scaffolding units in the striatin-interacting phosphatase and kinase (STRIPAK) complex [15] (Fig 1A). In mammals, this recently identified protein complex consists of striatin as scaffold, the putative kinase activator monopolar spindle-one-binder 3 (MOB3), serine/threonine-phosphatase PP2A subunits A and C, cerebral cavernous malformation 3 (CCM3) protein, the striatin-interacting protein (STRIP)1 and STRIP2 and the germinal center kinases (GCK) MST4, STK24, STK25 and MINK1 [15–19]. STRIPAK sub-complexes and STRIPAK-like complexes have since been identified in other organisms. In mammals, the STRIPAK sub-complex mutually exclusively interacts with the sarcolemmal membrane-associated protein (SLMAP), the related coiled-coil proteins suppressor of IKKepsilon (SIKE) and fibroblast growth factor receptor oncogene partner 2 (FGFR1OP2) or the cortactin-binding protein CTTNBP2 [15] (Fig 1A). In *Drosophila melanogaster* and mammals proteomic approaches identified the STRIPAK-like as a negative regulator of the Hippo signaling [20, 21]. The Hippo pathways consists of a kinase cascade that regulates tissue and organ size in metazoans [22] (Fig 1B). Core components of this pathways are highly conserved in the yeasts *Saccharomyces cerevisiae* and *Schizosaccharomyces pombe* as well as in filamentous fungi [23, 24]. In *S*. *cerevisiae* and fission yeast, one pathway is involved in coupling the cell cycle and cell septation and is termed the MEN (mitotic exit network) and SIN (septation initiation network), respectively [23, 25]. The second kinase pathway, which regulates morphology and polar growth is termed RAM (regulation of Ace2 and morphogenesis) in *S*. *cerevisiae* and MOR (morphogenesis of Orb6) in *S*. *pombe* [23, 26] (Fig 1B).

The STRIPAK-like complexes of fungi contain the striatin scaffolding unit, a STRIP1/2 homolog and PP2A subunits A and C [27, 31, 32, 36–39] (Fig 1A). They are designated factor arrest (FAR) complex in *S*. *cerevisiae*, SIN inhibitory PP2A (SIP) complex in *S*. *pombe* and STRIPAK-like complexes in filamentous fungi [28, 29, 31]. The STRIPAK-like complex of filamentous fungi contains in addition a homolog of MOB3, which is not encoded by *S*. *cerevisiae* and *S*. *pombe* (Fig 1A). However, fungal STRIPAK-associated kinases have not been characterized to date. In a previous study, we used a combined tandem affinity purification of PRO22, the tagged STRIP1/2 homolog of *S*. *macrospora*, followed by mass spectrometry using MudPIT (multidimensional protein identification technology) and identified the striatin homolog PRO11 and PP2A subunits A and C (PP2Ac1) as interaction partners, but surprisingly could not identify any GCK [31].

Mammalian GCKs regulate cellular processes, such as polarization, migration, cell growth, neuronal differentiation, apoptosis and stress responses [40, 41]. Fungal GCKs are involved in the regulation of cytokinesis, hyphal growth and differentiation of asexual development structures [42]. Based on structural similarity to the *S*. *cerevisiae* sterile-20 protein kinase (Ste20), GCKs are members of the Ste20-related group of protein kinases. These are sub-classified into p21-activated kinases (PAK) and GCK families based on the location of their kinase domain [43], which is C-terminal or N-terminal to the regulatory domain, in PAKs and GCKs, respectively [44]. GCKs share a highly conserved catalytic domain and a more divergent regulatory C-terminus [43, 44]. The GCK family can be further subdivided into subfamilies GCK I to GCK VIII [44].

In mammals, GCKs III MST4, STK24, STK25 and the GCK IV MINK1 are identified as components of the STRIPAK complex [15–17, 45]. CCM3 has recently been shown to act as an adaptor that recruits kinases of the GCK III family to the striatins [15, 17, 45]. The role of CCM3 is to bring GCK III into close proximity to striatin-connected phosphatase PP2A whereas GCK IV MINK1 is directly associated with striatin [16, 45]. It is thought that the PP2A phosphatase associated with the STRIPAK complex dephosphorylates the GCKs, and thereby reduces their catalytic activity [16, 45].

In order to identify STRIPAK kinases in *S*. *macrospora*, we performed a BLASTP search with the mammalian STRIPAK kinases MST4, STK24, STK25 and MINK1 against the *S*. *macrospora* genome. We identified two GCKs, SmKIN3 and SmKIN24, as possible homologs of the mammalian STRIPAK-associated GCKs. Y2H analyses revealed that both kinases could interact with the *S*. *macrospora* striatin homolog PRO11 and the interaction between SmKIN3 and PRO11 was verified *in vivo* by co-IP. Fluorescence microscopy revealed that both proteins localize to septa. Deletion of *Smkin3* led to fewer septa and increased protoplast regeneration and aerial hyphae formation, whereas deletion of *Smkin24* caused hyperseptation and sterility. Despite their distinct phenotypes, both kinases appear to act independently because the ΔSmkin3/ΔSmkin24 double-knockout exhibited a phenotype that combined those of the single-deletion strains.

## Materials and Methods

### Strains, media and culture conditions

All strains used in this study are listed in Table 1. Chemically competent *Escherichia coli* Mach1 was transformed according to a standard protocol [46]. Plasmids (Table A in S1 File) were generated by homologous recombination in yeast strain PJ69-4A [47] as described in [48] or with the In-Fusion HD Cloning Kit (Clontech, 639648). Transformation of yeast cells was performed by using an Eppendorf Electroporator 2510 (Eppendorf, Hamburg, Germany) at 1.5 kV [49]. *S*. *macrospora* transformation was done as described previously [50, 51] and selected on media supplemented with nourseothricin-dihydrogen sulfate (50 μg/ml) (WernerBioAgents, 5004000) or hygromycin B (110 U/ml) (Merck, 400051-10MU). *S*. *macrospora* strains (Tab 1) were grown in liquid or on solid corn meal (BMM) or fructification medium (Sordaria Westergaard) SWG [52, 53] at 27°C. Growth velocity of the respective strains was measured in 30 cm race tubes filled with 25 ml solid SWG medium. The average growth velocity represents the results of three independent experiments, each performed in triplicate.

**Table 1:** Strains used for this study.

Name	Genotype	Reference
***Sordaria macrospora***		
S48977	wild type	U. Kück, Bochum, Germany
S66001	Δ*ku70*::*nat* ^R^; fertile	[54]
S67813	mutation in gene *r2*; pink ascospores	[3]
ΔSmkin3	Δ*Smkin3*::*hyg* ^R^, ssi, fertile	this study
ΔSmkin3::pRS-Smkin3+	Δ*Smkin3*::*hyg* ^R^, pRS-Smkin3+^ect^, ssi, fertile	this study
ΔSmkin3::pDS-Smkin3ngfp	Δ*Smkin3*::*hyg* ^R^, pDS-Smkin3ngfp^ect^, ssi, fertile	this study
ΔSmkin24	Δ*Smkin24*::*hyg* ^R^, ssi, sterile	this study
ΔSmkin24::pRS-Smkin24+	Δ*Smkin24*::*hyg* ^R^, pRS-Smkin24+^ect^, ssi, fertile	this study
ΔSmkin24::pDS-Smkin24ngfp	Δ*Smkin24*::*hyg* ^R^, pDS-Smkin24ngfp^ect^, ssi, fertile	this study
ΔSmkin3/ΔSmkin24	Δ*Smkin3*::*hyg* ^R^, Δ*Smkin24*::*hyg* ^R^, ssi, sterile	this study
S48977::pHA11	nat^R^, pHA11^ect^, ssi, fertile	this study
S67813::pFLAG-SmKIN3	pink ascospores, hyg^R^, pFLAG-SmKIN3^ect^, ssi, fertile	this study
S48977:: pFLAG-SmKIN3, S48977::pHA11	nat^R^, hyg^R^, pFLAG-SmKIN3^ect^, pHA11^ect^, ssi, fertile	this study
***Saccharomyces cerevisiae***		
PJ69-4A	*MATa; trp1-901; leu2-3*,*112; ura3-52; his3-200; ga14Δ; ga18OΔ; LYS2*::*GALl-HIS3*: *GAL2-ADE2; met2*::*GAL7-lacZ*	[47]
Y187	*MATα; ura3-52; his3-200; ade2-101; trp1-901; leu2-3*,*112; gal4Δ; metΔ; gal80Δ; MEL1; URA3*::*GAL1*_*UAS*_ *-GAL1*_*TATA*_ *-lacZ*	Clontech, Mountain View, USA
AH109	*MATa; trp1-901; leu2-3*,*112; ura3-52; his3-200; ade2-101; gal4Δ; gal80Δ; LYS2*::*GAL1*_*UAS*_; *GAL1*_*TATA*_ *HIS3; GAL2*_*UAS*_ *GAL2*_*TATA*_ *-ADE2; URA3*::*MEL1*_*UAS*_ *-MEL1*_*TATA*_ *-lacZ; MEL1*	Clontech, Mountain View, USA
***Escherichia coli***		
Mach1	*ΔrecA139; endA1; tonA; Φ80*(*lacZ*)*Δ*M15; *ΔlacX74; hsdR(rK- mK+)*	Invitrogen, Germany

ect = ectopically integrated, FL = full length, nat = nourseothricin-resistance cassette, hph = hygromycin-resistance cassette, ssi = single- spore isolate, hyg^R^ = hygromycin resistant, nat^R^ = nourseothricin resistant

### Preparation of nucleic acids and PCR

Genomic DNA from *S*. *macrospora* was obtained as previously described in [4]. For PCR amplification Molzym MolTaq polymerase (Molzym GmbH & Co. KG, P-010-1000) or Phusion High-Fidelity DNA polymerase (New England Biolabs, M0530S) were used according to the manufacturer‘s protocol. All primers listed in Table B in S1 File were purchased at Eurofins MWG Operon (Ebersberg, Germany).

### RT-PCR

RNA from *S*. *macrospora* was isolated according to [55]. cDNA was generated via reverse transcription reaction using Transcriptor High Fidelity cDNA Synthesis Kit (Roche, 05081955001). Template concentration was adjusted to 2 μg RNA per reaction.

### Generation of *S*. *macrospora* knockout strains

Deletion strains were generated by transforming the respective deletion cassette into *S*. *macrospora* strain Δku70 which is impaired in non-homologous end joining [54]. For the construction of the deletion cassette, the 5’ and 3’ flanking regions of the respective genes were amplified via PCR with primers (Table B in S1 File) having 29-bp overhangs homologous to the hygromycin cassette as well as to the pRSnat or pRShyg plasmid backbone [31, 56]. The hygromycin resistance cassette *hph* was amplified from plasmid pCB1003 [57]. All fragments were merged and inserted into plasmid pRSnat or pRShyg by homologous recombination in the *S*. *cerevisiae* strain PJ69-4A [48]. This resulted in a deletion construct comprising the 5’ region, followed by *hph* and the 3’ region of the gene that should be deleted. The respective deletion constructs were entirely amplified via PCR, purified and transformed into *S*. *macrospora* Δku70. To eliminate the Δku70 (nourseothricin resistance) background *S*. *macrospora* primary transformants were crossed with spore-color mutant r2 [3]. Afterwards, hygromycin resistant, nourseothricin sensitive single-spore isolates were selected. Double-deletion strain ΔSmkin3/ΔSmkin24 was generated by crossing the single deletion strains ΔSmkin3 and ΔSmkin24. Deletion strains derived from single-spore isolates were verified by PCR using primers enlisted in Table B in S1 File and Southern hybridization using AlkPhos Direct Labelling and Detection Kit (GE Healthcare, RPN3690) [46].

### Protein extraction, Western blot analysis and co-Immunoprecipitation

Mycelium was grown for 3–5 days in liquid medium, dried and grounded in liquid nitrogen. Pulverized mycelium mixed with extraction buffer (100 mM Tris-HCl pH 7.6, 250 mM NaCl, 10% glycerol, 0.2% NP-40, 2 mM EDTA, 2 mM DTT) containing protease inhibitor cocktail IV (1:100, Roche, 04693132001) and 1 mM PMSF. Protein samples were separated by SDS-PAGE [58] and transferred onto nitrocellulose membranes by Western blot [59]. For immunodetection anti-FLAG (Sigma-Aldrich, F3165, 1:12000), anti-HA (Sigma-Aldrich, H9658, 1:3000) and secondary antibody anti-Mouse (Dianova, 115-035-003, 1:10000) were used. Anti-eGFP (Santa Cruz Biotechnology, sc-9996, 1:4000) antibody was used for expression control of eGFP-tagged kinases. Signals were detected using Immobilon Western Kit (Millipore, WBKLS0500).

### Co-immunoprecipitation

The plasmid encoding for the N-terminally HA-tagged PRO11 under control of the constitutive *N*. *crassa ccg1* promoter was described previously [31]. pFLAG-SmKIN3 encoding the N-terminally FLAG-tagged SmKIN3 was constructed by combining the full length cDNA of *Smkin3* (see version I of *Smkin3* transcripts Fig A in S1 File), with the *N*. *crassa ccg1* promoter of flowed by 3xFLAG [60, 61] and the *trpC* terminator of *Aspergillus nidulans* into vector pRShyg [31].

Plasmids encoding for HA-PRO11 [31] and FLAG-SmKIN3 were separately transformed into *S*. *macrospora* wt strain and combined by crossing resulting in a hygromycin and nourseothricin resistant strain expressing genes coding for both constructs. Protein purification was done as described above. The samples were separated by SDS-PAGE and blotted onto nitrocellulose membrane. For immunodetection, HA and FLAG antibody and HPR substrate were used as described above. The experiment was repeated two times independently. Co-IP was conducted with additional use of the thiol crosslinking reagent Bismaleimidohexane (BMH) (Thermo scientific, 22330) solved in Dimethylsulfoxide (DMSO = at a concentration of 20 mM. The crosslinker was used in a final concentration of 0.2 mM. The reaction was stopped after incubation for 2 h at 4°C by adding Dithiothreitol (DTT) in a final concentration of 25 mM as recommended in the manufacturers protocol.

### Yeast two-hybrid analysis

Y2H analysis in *S*. *cerevisiae* was conducted with the Matchmaker two-hybrid system 3 (Clontech, USA). pBD-SmKIN3 and pBD-SmKIN24 were generated by using In-Fusion HD Cloning Kit (Clontech, 639648). *Smkin3* (version with all introns spliced, version I in Fig A in S1 File) was amplified from cDNA with primers kin3_pBD_inf_F and kin3_pBD_inf_R, *Smkin24* (transcript version with all introns spliced, version I in Fig A in S1 File) was amplified from cDNA with primers kin24_pBD_inf_F and kin24_pBD_inf_R and inserted into vector pGBKT7. Plasmid pBD-SmKIN3 and pBD-SmKIN24 were transformed into yeast strain Y187, pAD11FL containing full-length *pro11* was transformed into AH109, respectively (Tab 1).

The respective Y187 and AH109 strains were mated as previously described (45) and selected on solid SD-medium lacking leucine (leu) and tryptophan (trp). Y187 and AH109 strains are adenine (ade), histidine (his), leu and trp auxotroph (Tab 1). Leu auxotrophy is restored with pGADT7 plasmid and derivatives, trp auxotrophy with pGBKT7 and derivatives. In AH109 the reporter gene *ade2* is under control of the GAL4 regulated *GAL2* promoter, but the gene coding for the GAL4 transcription factor is deleted (Tab 1). Proteins tested for interaction are fused either to GAL4 binding domain (BD) (vector pGBKT7) or GAL4 activation domain (AD) (vector pGADT7). Interaction of these proteins leads to close proximity of GAL4 DNA binding and activation domain and enable expression of the reporter gene. Growth of mated Y187-AH107 diploids on medium lacking leu, trp and ade, requires presence of both plasmids and interaction of the respective GAL4 fusion proteins. Positive colonies from SD-medium without leu and trp were used for drop plate assays. A serial dilution of cells was spread on SD agar plates without leu, trp and ade. To prove whether the respective genes in vector pGBKT7 were expressed properly, a test based on RanBPM was used by mating Y187 transformants with AH109 carrying pAD-RanBPM [62]. RanBPM interacts with the GAL4-binding domain and thus functions as expression control of the gene fused to GAL4 binding domain (54). pBD-SmKIN3 and pBD-SmKIN24 were also checked for self-activation as described in [63].

### Light and fluorescence microscopy investigations

To localize SmKIN3 and SmKIN24 in *S*. *macrospora*, N-terminally eGFP-tagged constructs under control of the *A*. *nidulans gpd* promoter were generated and introduced into their respective deletion strain. Since transcripts of *Smkin3* and *Smkin4* were alternatively spliced, plasmids encoding for SmKIN3-eGFP and SmKIN24-eGFP were constructed by amplifying the ORF of *Smkin3* or *Smkin24* corresponding to the version with all spliced introns of the transcript (version I in Fig A in S1 File). The cDNA was inserted into *Not*I digested pDS23-egfp vector [64] via homologous recombination in yeast. The respective primers, used for amplification of *Smkin3* (Smkin3ngfp_F x Smkin3ngfp_R) and *Smkin24* (Smkin24ngfp_F x Smkin24ngfp_R) are listed in Table B in S1 File. Hyphae, sexual structures and fluorescence were visualized by an AxioImager M1 microscope (Zeiss, distributed by Visitron Systems GmbH) combined with a Photometrics CoolSNAP_2 HQ_ camera (Roper Scientific, Photometrics). The respective pictures were processed with Metamorph (version 6.3.1; Universal Imaging), GIMP 2.8.2. (GNU Image Manipulation Program, The GIMP Development Team) and Illustrator CS2 (Adobe). To display eGFP or Calcofluor white fluorescence chroma filter set 49002 and 49000 were used, respectively. Calcofluor white staining for visualization of cell walls and septa was conducted by adding 40 μl Calcofluor white (Sigma-Aldrich, 18909), freshly diluted 1:1 with 10% KOH solution and directly applied on the mycelium.

### Phylogenetic analysis

Multiple protein sequence alignments were performed using the ClustalX2 program [65]. Phylogenetic analysis was made with programs from package PHYLIP version 3.695 (http://evolution.genetics.washington.edu/phylip.html). Distance matrices were calculated using the program PRODIST and were then used for constructing phylogenetic trees with the neighbor-joining (NJ) program NEIGHBOR. To evaluate the statistical significance a bootstrap analysis with 1000 iterations of bootstrap samplings and reconstruction of trees was performed. A majority rule consensus tree was subsequently generated with the program CONSENSE, displayed with TreeView 1.6.6 [66] and saved for graphical representation with Adobe Illustrator.

## Results

### *S*. *macrospora* encodes two kinases similar to mammalian STRIPAK-associated kinases STK24, STK25, MST4 and MINK1

In mammals, three members of the GCK III subfamily of the Ste20 kinases (STK24, STK25, and MST4); and one member of the GCK IV family (Misshappen-like kinase 1, MINK1) are associated with the STRIPAK complex [15, 16]. To date, no STRIPAK-associated kinases have been identified in filamentous fungi (Fig 1).

**Fig 2:**
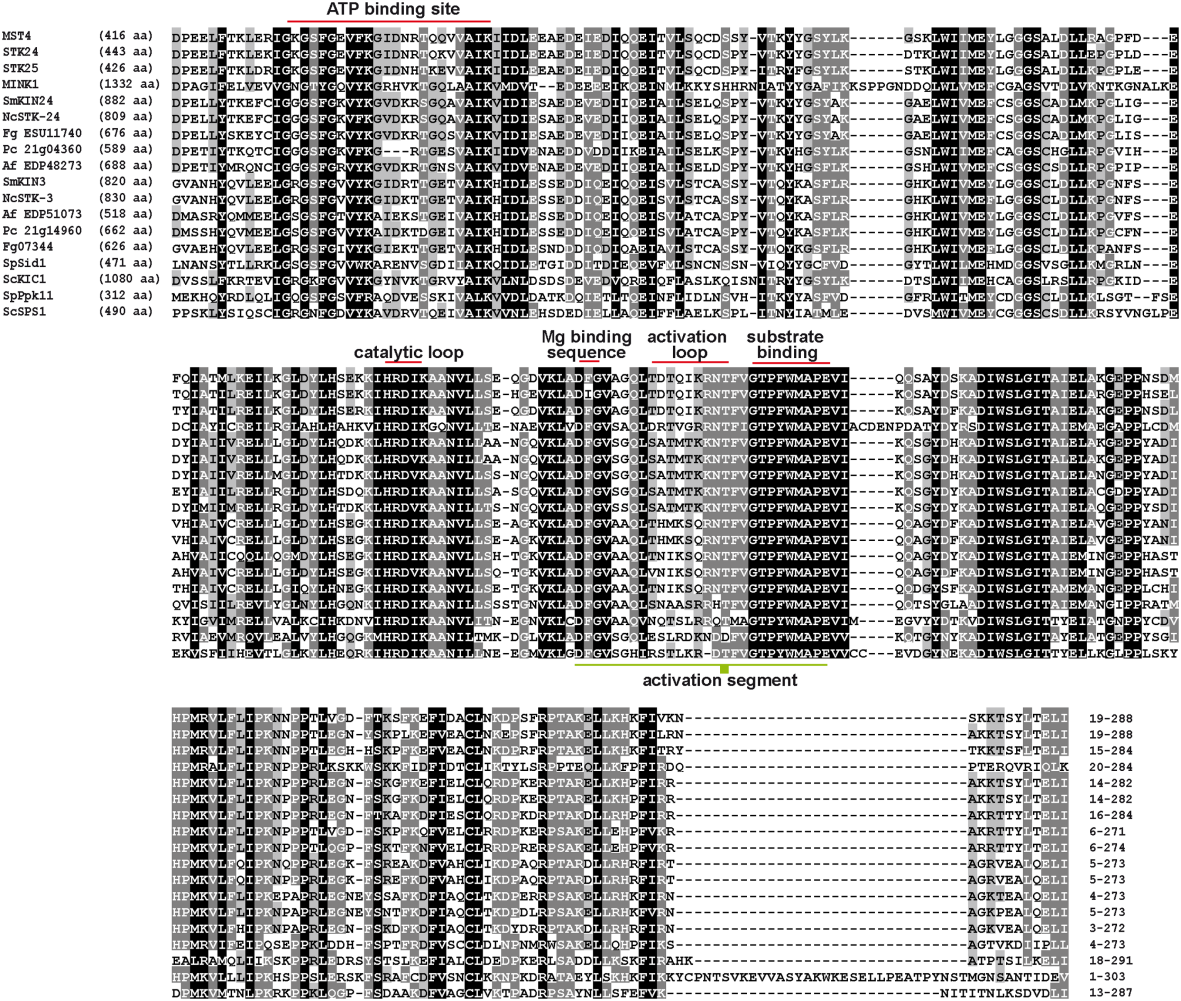
Multiple sequence alignment and amino-acid identity of mammalian kinases identified as STRIPAK members with putative homologs from Ascomycota. Protein sequences were aligned with ClustalX2 [65] and visualized with GeneDoc [70]. MST4, mammalian STE20-like protein kinase 4 (Accession number: Q9P289); STK24, mammalian STE20-like protein kinase 3 (Q9Y6E0); STK25, mammalian serine/threonine-protein kinase 25 (O00506); MINK1, mammalian misshapen-like kinase 1 (Q8N4C8); SmKIN24, putative serine/threonine-protein kinase 24 from *Sordaria macrospora* (F7VQV9), NcSTK-24 (STK-6, MST-1), serine/threonine-protein kinase 24 from *Neurospora crassa* (V5IQF9); Fg ESU11740, serine/threonine-protein kinase 24 from *Fusarium graminearum* (I1RNY7); Pc21g04360, putative STE20-like protein kinase from *Penicillium chrysogenum* (B6HMA9); Af EDP48273, putative STE20-like kinase from *Aspergillus fumigatus* (BA78_1793), SmKIN3, putative serine/threonine-protein-kinase 3 from *S*. *macrospora* (F7VYS5); NcSTK-3 (PRP-9, SID-1) serine/threonine-protein-kinase 3 from *N*. *crassa* (V5INC1); Af EDP51073, putative Ste20-like kinase from *A*. *fumigatus* (B0Y2A3); Pc21g14960, putative serine/threonine-protein kinase from *P*. *crysogenum* (B6HJ11); Fg07344, putative serine/threonine-protein kinase from *F*. *graminearum* (A0A016PPL3); SpSID1, serine/threonine-protein kinase from *Schizosaccharomyces pombe* (O14305); ScKIC1, serine/threonine-protein kinase from *Saccharomyces cerevisiae* (P38692); SpPPK11, serine/threonine-protein kinase from *S*. *pombe* (O14047); ScSPS1, sporulation-specific protein 1 from *S*. *cerevisiae* (P08458). Total numbers of amino acids are given in brackets. Regions of the aligned N-terminal domain of the protein sequences are indicated at the end of the alignment. ATP-binding side, catalytic loop, Mg-binding sequence, activation loop and region for substrate binding are marked with red lines. The entire activation segment is underlined in green. Filled box indicate the threonine residue (Thr190 in MST4, Thr182 in STK24, and Thr174 in STK25), phosphorylated during activation.

Therefore, we performed a BLASTP search using mammalian kinases STK24, STK25, MST4 and MINK1 as query against the *S*. *macrospora* proteome (http://blast.be-md.ncbi.nlm.nih.gov/ [67]) to identify putative *S*. *macrospora* homologs of the mammalian kinases. This search revealed the putative germinal center kinases SMAC_01456 (F7VQV9) and SMAC_04490 (F7VYS5) to be orthologous to the mammalian GCKs (Table 2). The predicted gene *SMAC_04490* comprises 2758 bp and contains three putative introns: 96 bp at position 53–148, 117 bp at position 217–333 and 82 bp at position 502–583 (Fig A in S1 File). The calculated molecular weight (MW) of the encoded 820-aa protein is 91.4 kDa with an isoelectric point (pI) of 9.47. It shows a high sequence identity to the serine/threonine-protein kinase 3 (*stk-3*) of *Neurospora crassa* (locus tag NCU04096, gene symbol *prk-9* alias *sid-1*) (Fig 2) and was therefore named SmKIN3. Two variants have been reported for the *N*. *crassa* NCU04096 protein: a long version resulting from a transcript after splicing of three introns, and a short version derived from a transcript retaining intron 3 and following initiation of translation at a downstream ATG codon (http://www.broadinstitute.org/annotation/genome/neurospora/). The position of all three introns is conserved in *Smkin3* and *stk-3* (*prk-9*, *sid-1)*, but we identified splicing of all three introns in *Smkin3* using RT-PCR and cDNA sequencing, and splicing of intron 3 did not appear to be optional (Fig A in S1 File). In addition, the position of the downstream start codon is conserved in *Smkin3*. Initiation of translation from this ATG codon would lead to an N-terminally truncated SmKIN3 version of 663 aa with an MW of 74 kDa (Fig A in S1 File).

**Table 2:** BLASTP search of the human STRIPAK associated GCKs against the *S*. *macrospora* proteom.

Type	*Homo sapiens* (accession number)	*S*. *macrospora* best hit (e-value)
GCK III	MST3, STK24 (Q9Y6E0.1)	SMAC_01456 (5e-136) SMAC_04490 (9e-126)
GCK III	MST4, MASK (Q9P289.2)	SMAC_01456 (4e-136) SMAC_04490 (6e-125)
GCK III	STK25, SOK1, YSK1 (O00506.1)	SMAC_01456 (3e-134) SMAC_04490 (4e-123)
GCK IV	MINK1 (NP_722549.2)	SMAC_01456 (8e-75) SMAC_04490 (2e-69)

The second predicted gene *SMAC_01456* encoding a putative homolog of mammalian GCKs III and GCKs IV, encompasses 2947 bp and is predicted to be disrupted by four introns: 107 bp at position 77–183, 76 bp at position 215–290, 51 bp at position 608–658 and 64 bp at position 2413–2476. The calculated MW of the encoded protein of 882 aa is 98.3 kDa with an isoelectric point (pI) of 7.70. The closest homolog of this protein is serine/threonine-protein kinase 24 (*stk-24*) of *N*. *crassa* (locus tag NCU00772, gene symbol *stk-6* alias *mst-1*). The *S*. *macrospora* protein was therefore designated SmKIN24. Similarly to *NCU04096*, two gene products have been identified for *NCU00772*: a long version resulting from splicing of three introns at same positions as the first three introns of *Smkin24*, and a short version resulting from skipping of intron 1 and translation initiation at a downstream ATG start codon. This downstream start codon is conserved in *Smkin24*. RT-PCR and cDNA sequencing of *Smkin24* revealed that, like *NCU00772*, splicing of the first intron was optional. Additionally, sequencing of full length RT-PCR products showed optional splicing of intron 4, which is not present in *NCU00772* (Fig A in S1 File). Thus, in addition to the full-length protein two further variants are encoded by *Smkin24*. Skipping the splicing of intron 1 and initiation of translation at the downstream ATG codon would result in a 789 aa protein with a MW of 88 kDa, whereas skipping intron 4 splicing would lead to a C-terminal truncated version of 729 aa with a MW of 82 kDa (Fig B in S1 File).

In mammalian kinases of GCK III and IV family the regulatory domain lies C-terminal to the catalytic domain and is heterogeneous in sequence [68]. A search for conserved domains at CCD http://www.ncbi.nlm.nih.gov/Structure/cdd/wrpsb.cgi [69] revealed that both SmKIN3 and SmKIN24 exhibit a typical STKc MST3-like catalytic domain of mammalian Ste20-like kinase 3 serine/threonine-protein kinases (accession cd06609, e-value SmKIN3 = 1.13e^-177^, e-value SmKIN24 = 0.0) at the N-terminal part of the protein. As shown in Fig 2, SmKIN3 and SmKIN24 possess a conserved ATP-binding site [GXGX(F)GX_16_ K], a catalytic loop sequence [HRDIK], a Mg^2+^-coordinating sequence [DFG], a substrate binding motif [GTPFWMAPE] and a critical threonine residue, phosphorylated during activation, at the end of the activation loop [40, 41]. Based on an aa alignment of the N-terminal catalytic domain, *S*. *macrospora* SmKIN3 and SmKIN24 share a high level of sequence identity with the N-terminus of the mammalian MST4 (67%/67%), STK24 (68%/68%), STK25 (67%/68%) and MINK1 (46%/46%) (Fig C in S1 File).

**Fig 3:**
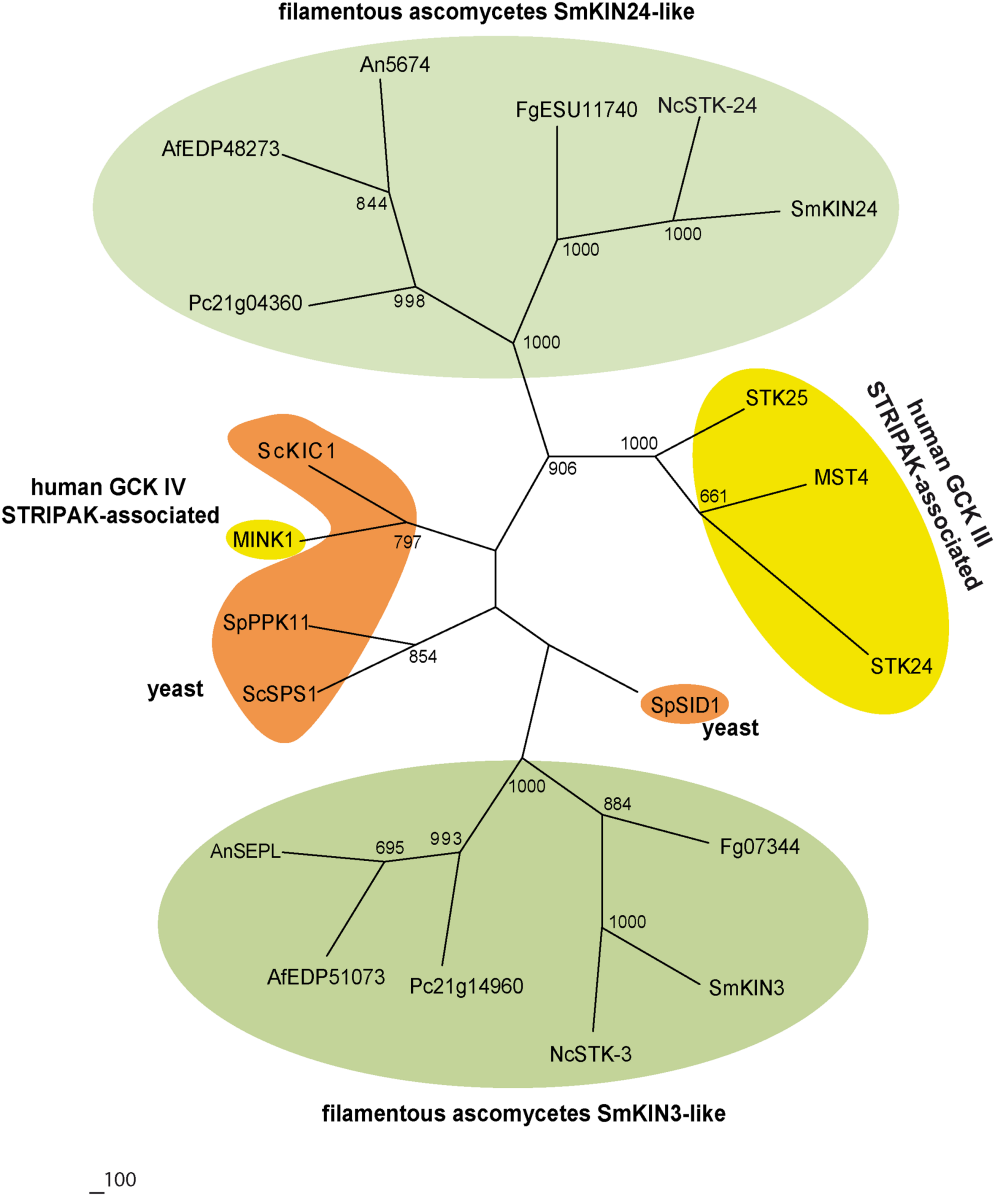
Unrooted neighbor joining tree of human GCKs MST4, STK24, STK25, MINK1 and their orthologues in ascomycetes. Catalytic domains of the proteins were aligned with ClustalX2 [65]. The human GCKs are displayed in yellow, the orthologues from the yeast *S*. *pombe* and *S*. *cerevisiae* in orange. SmKIN3-like kinases and SmKIN-24-like kinases from filamentous ascomycetes are shown in green. Accession numbers of the proteins are given in Fig 2 except of the *Aspergillus nidulans* GCKs AnSEPL (C8V5Z7) and An5674 (Q5B1A6). The number at the nodes indicates bootstrap support of 1000 iterations.

However, the C-terminally located regulatory domains are structurally different to GCKs from other filamentous ascomycetes, as well as *S*. *cerevisiae* and *S*. *pombe* (less than 20%). Phylogenetic analysis revealed that SmKIN3 and SmKIN24 were strictly separated and cluster together with putative homologs from other filamentous ascomycetes (Fig 3).

Mammalian GCK III members contain putative nuclear export signals (NES) and bipartite nuclear localization sequences (NLS) [71]. A putative NES at aa position 493–509 (LSDAFAHLDL) was predicted for SmKIN3 by NetNES1.1 [72] and a bipartite NLS by the programme cNLS [73] for SmKIN3 at position 463–490 (RRLYNKAIEPALEELHAQTGPSQKREAL) (score 4.1) and SmKIN24 at position 784–817 (EEALKRRQIMLQQTYRPEPGYAPSPPTPKQQRAE) (score 3.8).

### SmKIN3 and SmKin24 interact physically with PRO11

**Fig 4:**
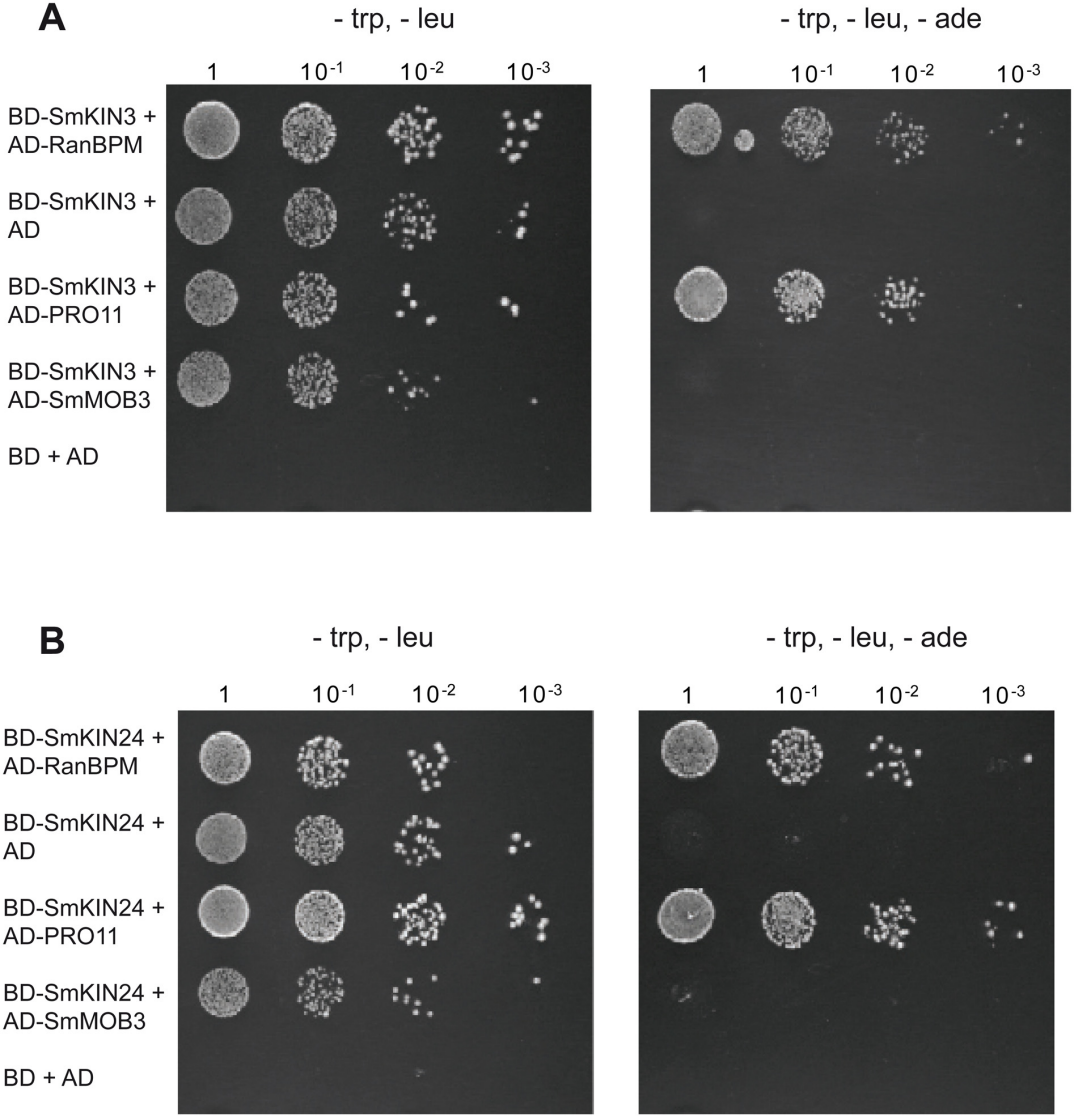
PRO11 interacts physically with SmKIN3 (A) and SmKIN24 (B) in a yeast two-hybrid analysis. Shown are serial dilutions of diploid yeast strains obtained after mating and spread on SD medium lacking tryptophan (trp) and leucine (leu) or trp, leu and adenine (ade) to verify the interaction of both proteins. The left picture displays a control, which ensures presence of both plasmids in the diploid strain; trp and leu prototrophy are regained by genes present on the plasmid. The right picture displays the interaction assay; ade prototrophy is obtained by positive interaction between the proteins fused to GAL4 binding domain (BD) and GAL4 activation domain (AD). Full length versions of SmKIN3 and SmKIN24 without any introns were tested. GAL4-binding domain was fused to SmKIN3 or SmKIN24 GAL4 activation domain to PRO11. Reverse application of activation and binding domain was not possible due to transactivation of BD-PRO11. Yeast transformants expressing genes coding for BD-SmKIN3 or BD-SmKIN24 and AD-RanBPM served as positive control (46). As negative control a diploid strain carrying empty vectors pGADT7 (AD) and pGBKT7 (BD) was used. No interaction between SmMOB3 and SmKIN3 or SmKIN24 has been observed.

Striatin is the scaffold of mammalian STRIPAK complex kinases MST4, STK24, STK25 and MINK1 [15, 16]. PRO11 was previously shown to be the *S*. *macrospora* homolog of mammalian striatin [8]. The sequence similarity between SmKIN3 and SmKIN24 and the mammalian kinases of the GCK III and GCK IV family (Fig 1) led us to inspect whether both *S*. *macrospora* kinases can interact with PRO11 in a Y2H system. Full-length cDNAs (version I with all introns spliced, Fig A in S1 File) of *Smkin3* and *Smkin24* were cloned into the Y2H vector pGBKT7 that contains the GAL4 DNA-binding domain. The pGADT7-derived plasmid pAD11FL, which encodes a full-length PRO11 fused to the GAL4 activation domain, served as the prey vector. Plasmids were transformed into yeast strains Y187 (pGBKT7 constructs) or AH109 (pGADT7 constructs). Strains carrying pBD-SmKIN3 and pBD-SmKIN24 plasmids were checked for transactivation activity by mating with yeast strain AH109 containing pGADT7. A strain carrying pGBKT7 and pGADT7 was used as negative control. Gal4 fusion proteins of SmKIN3 and SmKIN24 were checked for adequate expression and two-hybrid competency using the RanBPM system [62]. Previously, we showed that the MOB domain protein SmMOB3, the mammalian phocein homologue, is a strong interaction partner of PRO11 [31, 74]. We therefore tested the interaction of both kinases with SmMOB3. Using the Y2H system we confirmed that both SmKIN3 and SmKIN24 could physically interact with PRO11 but not with SmMOB3 (Fig 4 and Fig D in S1 File).

**Fig 5:**
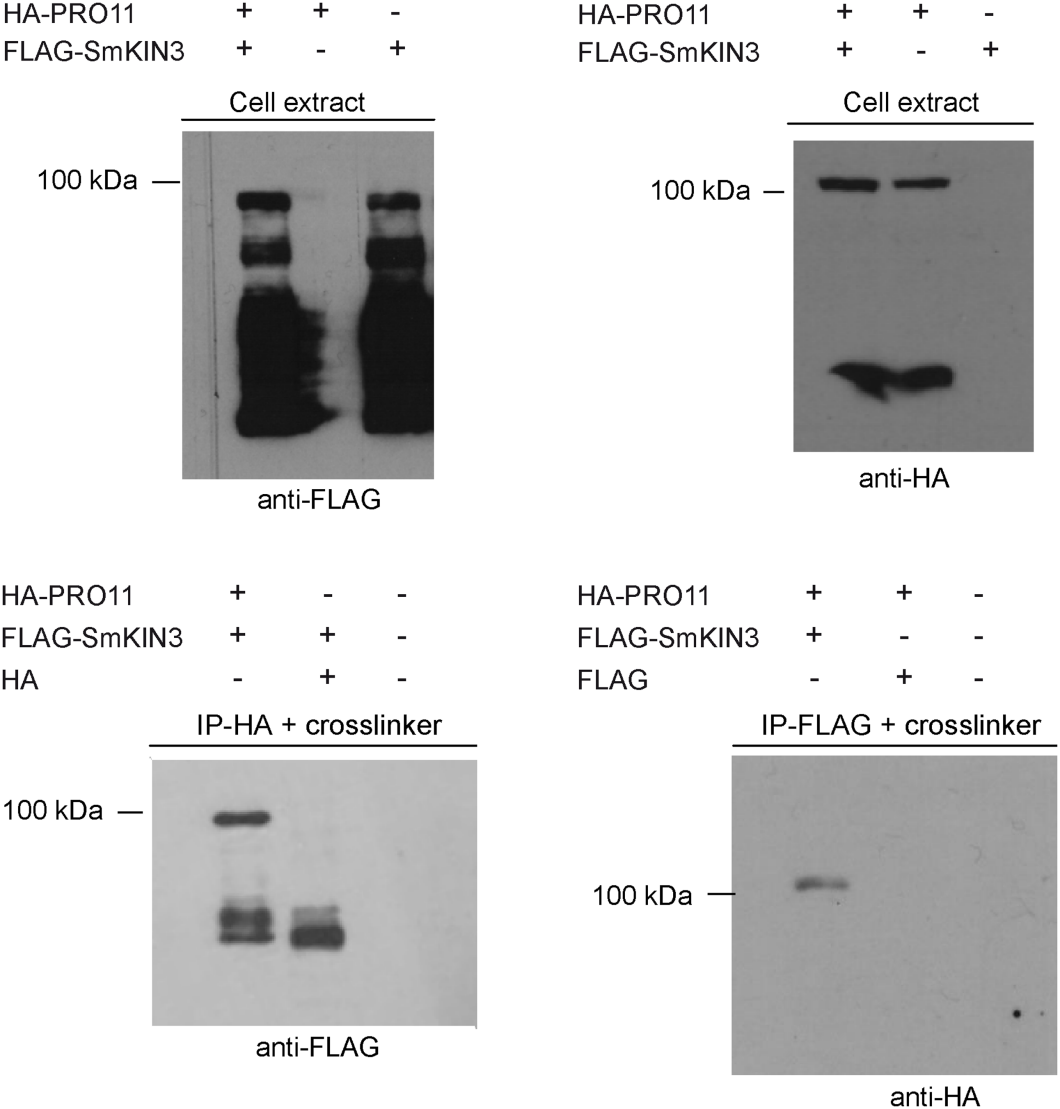
Co-immunoprecipitation of HA-PRO11 and FLAG-SmKIN3 visualized by Western blot analysis. The strain used for co-IP expressed the respective proteins in suitable amounts (cell extracts); HA-PRO11 shown by anti-HA antibody (anti-HA) and FLAG-SmKIN3 detected by FLAG antibody (anti-FLAG). FLAG-SmKIN3 was pull-downed with HA-PRO11 as bait and identified by Western blot using anti-FLAG antibody (IP-HA). For this experiment, a strain expressing FLAG-SmKIN3 and the HA tag without any fused protein served as negative control and was treated equivalent to the co-IP sample. To exclude unspecific antibody binding, protein extract of the wt was used as second negative control for Western blot analysis. HA-PRO11 was isolated using FLAG-SmKIN3 as bait and identified by Western blot (IP-FLAG) using an anti-HA antibody. A strain expressing genes coding for the FLAG-tag without any fused protein and HA-PRO11 served as negative control and was treated as the co-IP sample. To exclude unspecific antibody binding, protein extract of the wt was used as second negative control for Western blot analysis. “+” mark strains expressing the respective construct.

To test whether PRO11 and GCKs SmKIN3 and SmKIN24 could interact *in vivo*, we performed co-IP studies in *S*. *macrospora*. We expressed N-terminally FLAG-tagged SmKIN3 and HA-tagged PRO11 [31] in *S*. *macrospora*. We were not able to express FLAG-tagged SmKIN24 in *S*. *macrospora* wt in amounts suitable for co-IP, under control of either the native or the strong, constitutive *ccg1* promoter of *N*. *crassa* [61]. Expression of *Smkin24* in the ΔSmkin24 deletion background was possible in low amounts suitable for localization (see below) but expression in wt for co-IP was necessary since only two resistance markers exist and no recombinase-mediated excision system is yet established for *S*. *macrospora*. All obtained viable transformants expressed *Smkin24* in low amounts. We therefore concluded that SmKIN24 is highly unstable. However, tagged SmKIN3 and PRO11 could be expressed separately and co-expressed in wt *S*. *macrospora*. Pull-down experiments without adding a crosslinker did not result in precipitation of PRO11 (Fig E in S1 File). Since protein kinases often interact weakly and transiently with their substrates, we performed sulfhydryl crosslinking with BMH which confirmed the physical interaction of full length SmKIN3 and PRO11 (Fig 5).

### Deletion of *Smkin3* or *Smkin24* impairs vegetative growth but only ΔSmkin24 is sterile

**Fig 6:**
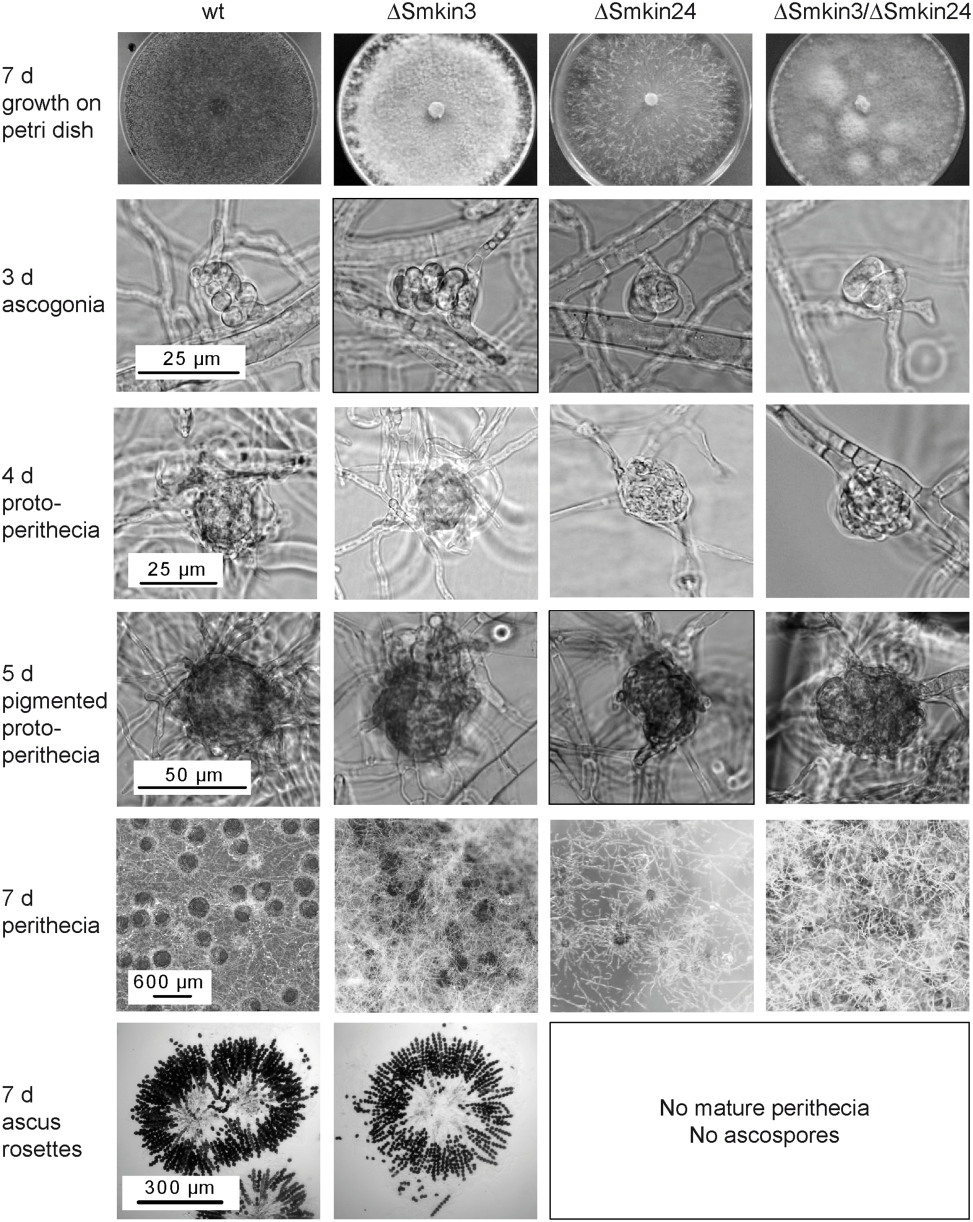
Macroscopic and microscopic analysis of the sexual development of wt, ΔSmkin3, ΔSmkin24 and ΔSmkin3/ΔSmkin24. The wt strain produces ascogonia after 3 days which develop to unpigmented protoperithecia at day 4 and pigmented protoperithecia at day 5. After 7 days mature perithecia with asci and ascospores are formed. Like the wt, ΔSmkin3 completes the lifecycle within 7 days and produces germinable ascospores. Development of ΔSmkin24 and the double-deletion strain ΔSmkin3/ΔSmkin24 is arrested at the stage of late protoperithecia formation. ΔSmkin3 produces more arial hyphae than the wt and all perithecia are embedded in a layer of whitish mycelium at day 7 of development. Scale bars as indicated.

In order to investigate the functional role of *Smkin3* and *Smkin24*, we generated single- and double-deletion strains ΔSmkin3, ΔSmkin24 and ΔSmkin3/ΔSmkin24 (Fig F and Fig G in S1 File). The respective genes were replaced with a hygromycin resistance cassette by homologous recombination in the *S*. *macrospora* Δku70 [54] strain (Fig F and Fig G in S1 File). The Δku70 background was removed afterwards by crossing the homokaryotic deletion strains with *S*. *macrospora* spore-color mutant r2 [3] and the obtained single-spore isolates were verified by PCR and Southern blotting (Fig F and Fig G in S1 File). Microscopic analysis of the knockout strains revealed that only ΔSmkin3 was fertile and able to generate fruiting bodies with ascospores within 7 days (Fig 6). The deletion strain ΔSmkin24 was halted during late protoperithecia maturation and thus failed to develop mature fruiting bodies (Fig 6). Additionally, ΔSmkin3 produced more aerial hyphae on solid SWG fructification medium compared to wt (Fig 6). The double-deletion strain was generated by crossing the single deletion strains, and ΔSmkin3/ΔSmkin24 exhibited a phenotype that was a combination of the phenotypes of both single-deletion strains (sterility and increased aerial hyphae on solid SWG medium). Each of the described phenotypes could be complemented by inserting a copy of the deleted gene ectopically (Fig H in S1 File). Sterility of STRIPAK mutants pro11, pro22, Δpro45 and ΔSmmob3 is accompanied by defects in hyphal fusion [31, 32, 74]. However, in contrast with these previously characterized mutants, fusion of vegetative hyphae was not affected in ΔSmkin3 and ΔSmkin24 (Fig I in S1 File).

Furthermore, deletion of *Smkin3* and *Smkin24* impaired vegetative growth of *S*. *macrospora*: Growth rate experiments revealed that ΔSmkin3 (1.70 ± 0.24 cm/d), ΔSmkin24 (1.82 ± 0.41 cm/d) and ΔSmkin3/ΔSmkin24 (1.54 ± 0.28 cm/d) grew more slowly than wt (3.09 ± 0.37 cm/d).

### SmKIN3 and SmKIN24 localize to septa and influence septum formation

**Fig 7:**
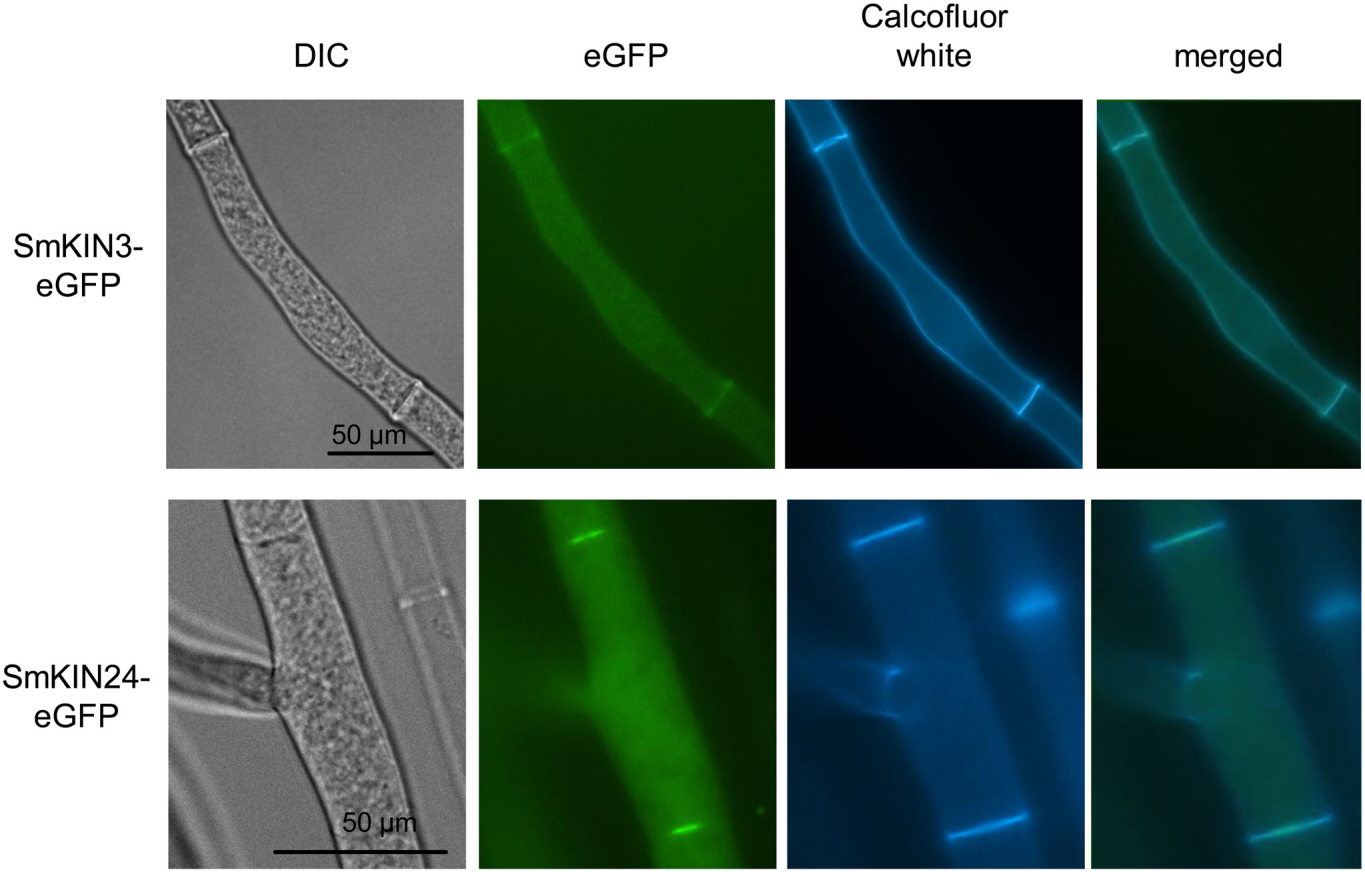
Localization of SmKIN3-eGFP and SmKIN24-eGFP in *S*. *macrospora*. SmKIN3-eGFP and SmKIN24-eGFP localize to septa. For visualization of cell walls and septa, hyphae were co-stained with Calcofluor white. DIC = differential interference contrast microscopy, scale bars as indicated.

**Fig 8:**
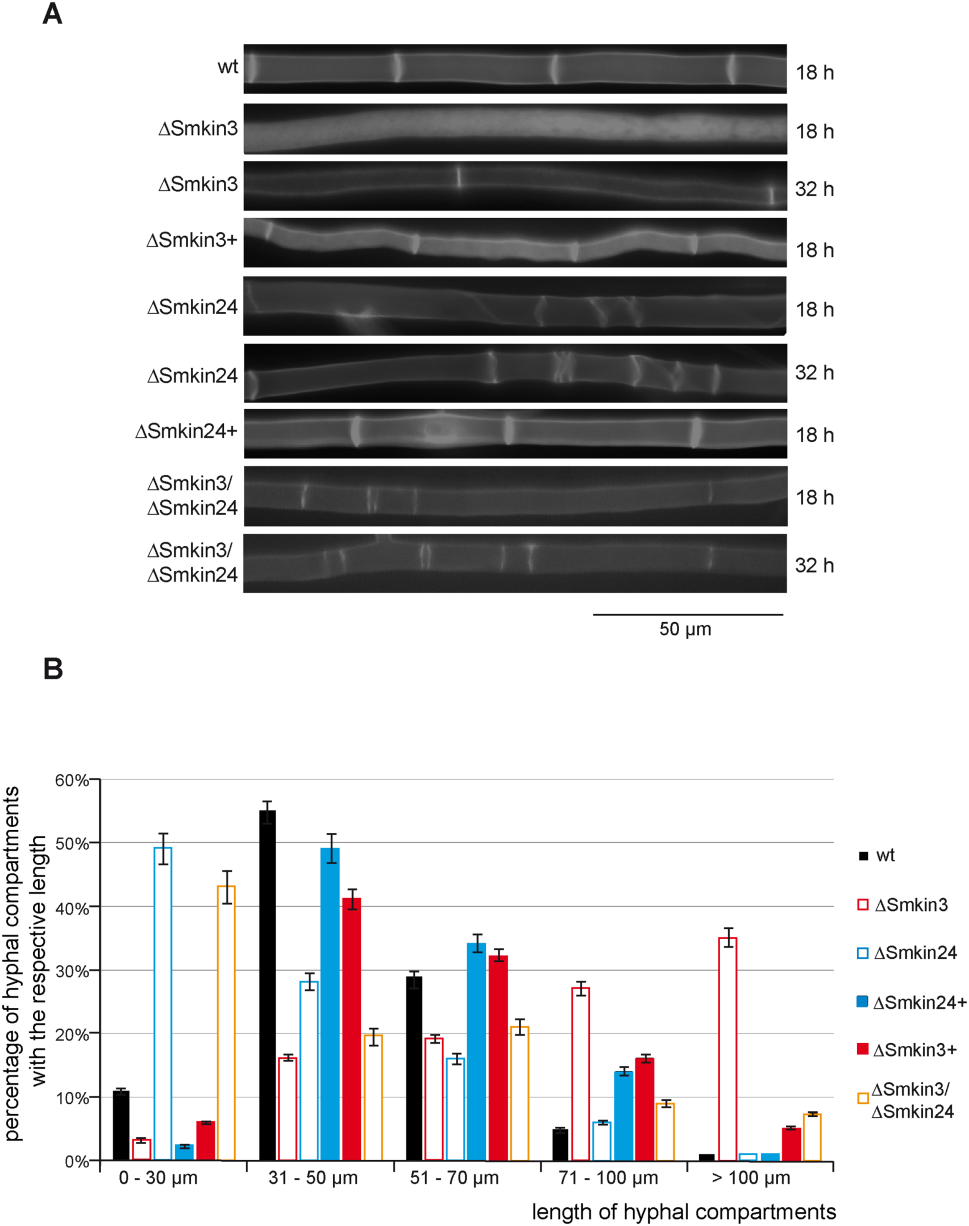
Analysis of septal development in wt, ΔSmkin3, ΔSmkin24 and ΔSmkin3/ΔSmkin24 and complemented mutants. (**A**) Distribution of septa was investigated under the microscope after 18 h and 32 h past inoculation. Septa were stained with Calcofluor white. Scale bar as indicated. (**B**) For quantification, distances between adjacent septa were measured over a distance of 18 mm per strain 24 h past inoculation. The total distance of 18 mm was divided into 30 segments of 600 μm. Measurements were binned into five different compartment lengths: 0–30 μm, 31–50 μm, 51–70, 71–100 μm, and more than 100 μm. The number of analyzed compartments was normalized to 100%. Error bars (SD) are given as indicated (n = 30). ΔSmkin3+, ΔSmkin24+ complemented mutants carrying an ectopic copy of the respective wt gene.

In order to determine the localization of SmKIN3 and SmKIN24 *in vivo*, fluorescence microscopy was performed. Since C-terminally tagged versions of SmKIN3 and SmKIN24 did not complement the mutant phenotype, genes encoding N-terminally eGFP-tagged full-length SmKIN3 or SmKIN24 were expressed in the respective *S*. *macrospora* deletion strains. These constructs complemented the mutants and revealed that both kinases were localized at the septa (Fig 7). However, SmKIN3 was localized mainly at the outer part of the septum, whereas SmKIN24 was localized at the septal pore (Fig 7), and this was verified by co-staining with Calcofluor white (Fig 7). These findings lead us to quantify septum formation in the ΔSmkin3, ΔSmkin24 and ΔSmkin3/ΔSmkin24 strains and their respective complementation strains by staining with Calcofluor white after 18 or 32 h of growth (Fig 8A). Furthermore, we quantified the distances between adjacent septa in wt and deletion mutants after 24 h of growth (Fig 8B). Septa were distributed in a uniform manner in wt with hyphal compartment length between 31–70 μm (Fig 8B). In contrast, ΔSmkin3 exhibited larger distances between adjacent septa (>71 μm), although this effect begun to revert after 24 h of growth (Fig 8A). Strikingly, ΔSmkin24 developed numerous closely-packed septal bundles of abnormal shape, with much smaller hyphal compartments of 0–30 μm (Fig 8B). ΔSmkin3 produced about half of the total number of septa within a distance of 18 mm than wt, while ΔSmkin24 produced 20% more than wt after 24 h of growth. With respect to septation, the double-deletion mutant ΔSmkin3/ΔSmkin24 displayed a similar phenotype to the ΔSmkin24 single-deletion strain. Ectopic integration of the respective wt gene into deletion mutants restored septal formation to wt level (Fig 8A and 8B). The septa of ΔSmkin3 and ΔSmkin24 were checked for functionality under the microscope directly after cutting of the hyphae. Both strains did not differ from wt; shortly after cutting, hyphal compartments of ΔSmkin3 and ΔSmkin24 were plugged by Woronin bodies.

### ΔSmkin3 protoplasts recover significantly faster than wt protoplasts

**Fig 9:**
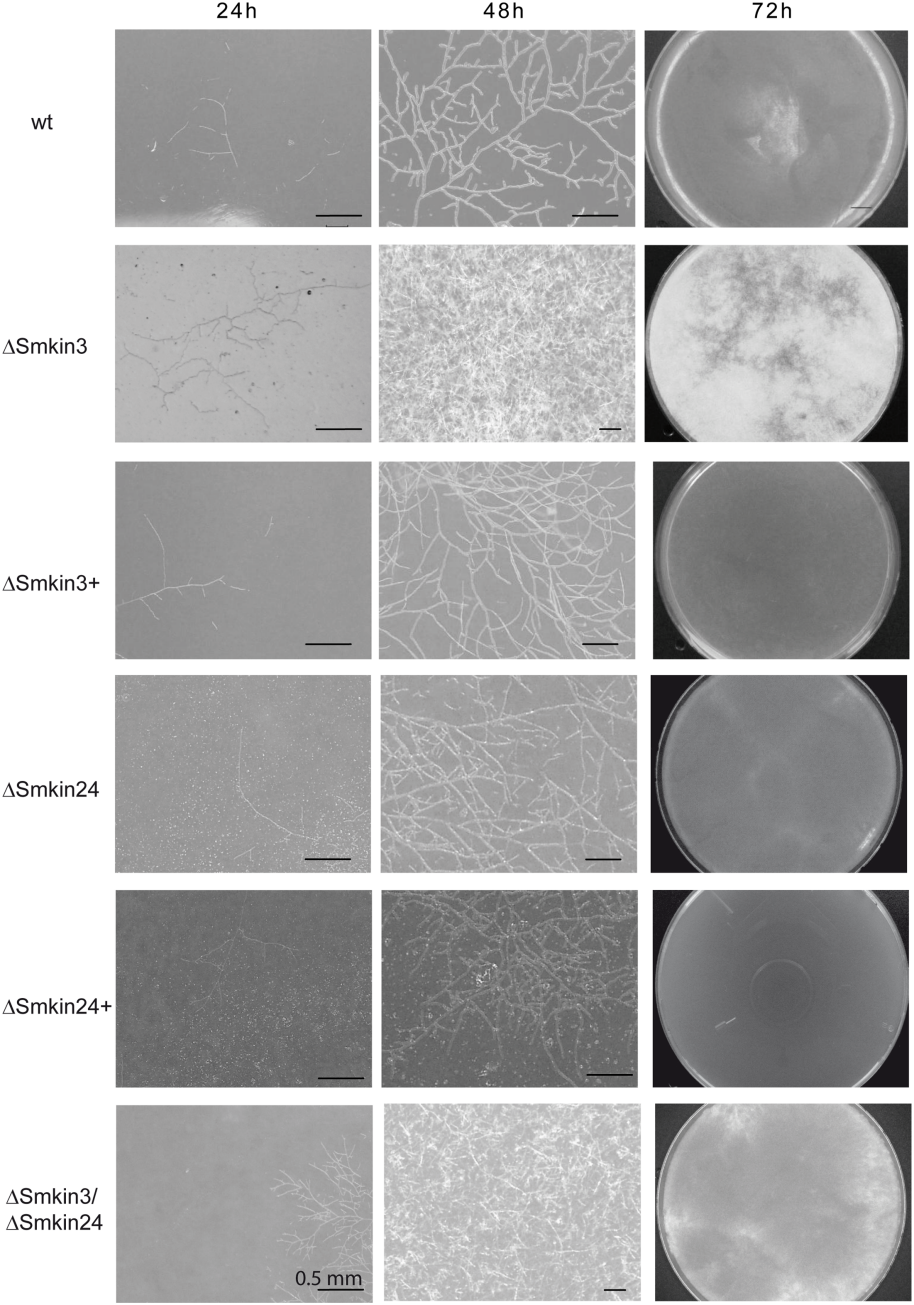
Investigation of protoplast recovery and vegetative growth of ΔSmkin3, ΔSmkin24, the respective complemented strains and ΔSmkin3/ΔSmkin24. Protoplasts of the respective strains were obtained as described in [50] and spread on solid CMS agar plates. Microscopic pictures were taken after 24 h, 48 h past inoculation, pictures of agar plates 72 h past inoculation. Compared to wt, protoplasts obtained from ΔSmkin3 and ΔSmkin3/ΔSmkin24 recovered and grew faster within the first 2–3 days. Recovery and vegetative growth of protoplasts obtained from ΔSmkin24 is equal to wt. ΔSmkin3+, ΔSmkin24+ complemented mutants carrying an ectopic copy of the respective wt gene. Scale bars as indicated.

We observed an increased growth rate of protoplasts from mutant ΔSmkin3 compared to wt protoplasts, therefore we isolated protoplasts from wt, and strains ΔSmkin3, ΔSmkin24 and ΔSmkin3/ΔSmkin24 and their respective complementation strains using a previously described protocol [50, 51] and adjusted the protoplast concentration to 4 x 10^4^ protoplasts/ml. The protoplasts were spread on solid complete medium with 10.8% saccharose (CMS) [50] and microscopically and macroscopically analyzed after 24, 48 and 72 h of growth (Fig 9). After 24 h, wt protoplasts developed a slightly branched mycelium, which expanded further by 48 h (Fig 9). Mycelia were only faintly visible without magnification on agar plates after 72 h. Regeneration of protoplasts from strain ΔSmkin24 resembled those of wt. In contrast, protoplasts from mutants ΔSmkin3 and ΔSmkin3/ΔSmkin24 recovered markedly faster within the first days than wt and mycelia generated by mutant ΔSmkin3 protoplasts were much denser and clearly visible with the naked eye after 72 h. Increased aerial hyphae were also present in mutant ΔSmkin3 and this effect was complemented by ectopically integrated wt copy of *Smkin3* in the ΔSmkin3 mutant (Fig 9). The accelerated growth rate of ΔSmkin3 vegetative mycelium disappeared after five days. The increase distance between adjacent septa of mutant ΔSmkin3 could also cause larger protoplasts which are more likely to recover. Therefore, we measured the diameter of protoplasts from wt, mutants ΔSmkin3 and ΔSmkin24, but observed no differences between all strains. Moreover, addition of the cell wall disturbing drug Calcofluor white did not affect the increased growth rate of hyphae derived from ΔSmkin3 protoplasts.

## Discussion

In mammals, GCKs are implicated in fundamental developmental processes, such as proliferation, cell polarity and cell migration [40, 75–77]. Previously, it was shown that the GCKs MST4, STK24, STK25 and MINK1 bind to striatin and therefore are part of the mammalian STRIPAK complex [15, 16]. The STRIPAK core components striatin and phosphatase PP2A are conserved from yeast to man [37] (Fig 1A). The *S*. *macrospora* STRIPAK complex contains the striatin homologs PRO11 and PP2A catalytic and scaffold subunits as well as the STRIP1/2 homolog PRO22, the SLMAP homolog PRO45 and the phocein homolog SmMOB3 [31, 32]. However, until now, evidence for the presence of STRIPAK-related kinases in ascomycetes was lacking. Here, we analyzed putative GCKs in *S*. *macrospora* and addressed their relevance as putative STRIPAK-associated kinases.

### Are SmKIN3 and SmKIN24 STRIPAK-associated kinases?

A BLASTP search identified SmKIN3 and SmKIN24 as putative homologs of mammalian STRIPAK kinases. The N-terminal part of the mammalian STRIPAK kinases, containing the kinase domain, is conserved among ascomycetes (Fig 2). Moreover, the phylogenetic analysis of MST4, STK24, STK25 and MINK1 and their orthologs in ascomycetes revealed two groups of putative STRIPAK-associated GCKs in filamentous fungi (Fig 3).

SmKIN3 and SmKIN24 interacted with PRO11 in Y2H (Fig 4A and 4B) and co-IP experiments (SmKIN3 with PRO11) (Fig 5). However, successful co-IP results required crosslinking, indicating weak interaction. In *N*. *crassa*, the GCK STK-3 (PRK-9, SID-1; homolog of SmKIN3) is part of SIN/MEN, whereas STK-24 (STK-6, MST-1; homolog of SmKIN24) has a dual role in SIN/MEN and MOR/RAM (Fig 1B) [35, 78]. SIN/MEN is a signal transduction pathway required for proper coordination of mitosis and cytokinesis [23, 25]. Following cytokinesis MOR is essential for cell polarity control and septum formation [23, 79, 80]. Both *N*. *crassa* kinases are regulated by the upstream kinase CDC-7, however in an opposite manner (Fig 1B)[35].

*N*. *crassa* STK-3 (PRK-9, SID-1) and STK-24 (STK-6, MST-1) both localize to the septa and spindle pole bodies (SPB) [35, 78]. In *S*. *pombe*, SIN components bind to the coiled coil scaffold protein SID4 [81]. However, a clear homolog of the SID4 has not been identified in ascomycetes [78]. Based on interaction studies, STRIPAK PRO11 may serve as a scaffold for SmKIN3 and SmKIN24 and we therefore predict interplay between STRIPAK and SIN/MEN in *S*. *macrospora*. This hypothesis is supported by findings in fission yeast, where the STRIPAK-like complex is called SIP complex (Fig 1A) [28]. The fission yeast SLMAP-homolog CSC1 was shown to negatively regulate SIN and promote SIN asymmetry [28] which is crucial for cytokinesis. In *D*. *melanogaster*, STRIPAK components negatively regulate Hippo signaling [20], which is homologous to the fungal SIN/MEN and RAM/MOR networks [23, 82] (Fig 1B). The Hippo pathway is a central element of tissue size control in *D*. *melanogaster* and higher organisms. It consists of an upstream kinase Hpo (MST1/2 in mammals), downstream NDR kinases LATS1/2 and NDR1/2 associated MOB family proteins and various scaffolding subunits [23, 83, 84] (Fig 1B). Based on interaction of Hpo/MST1/2 with homologs of MOB3 and striatin, the *D*. *melanogaster* dSTRIPAK complex was identified as the negative regulator of Hippo signaling. Loss of dSTRIPAK components resulted in elevated phosphorylation of Hpo [20]. Similarly, in mammals it was demonstrated that the Hippo kinases MST1 and MST2 bind the SLMAP-STRIPAK complex [21]. A BLASTP analysis with Hpo, MST1 and MST2 protein sequences revealed SmKIN3 and SmKIN24 as orthologs of these kinases in *S*. *macrospora*. However, SmKIN3 and SmKIN24 did not interact with SmMOB3 in Y2H experiments (Fig 2). In *N*. *crassa*, a direct interaction of STK-24 (SID-1) and the NDR kinase DBF-2 was identified by MS and yeast two-hybrid studies; however an interaction with the kinase activator MOB-1 was not tested. It may be therefore meaningful considering interactions of other MOB-family proteins and STRIPAK associated GCKs to define the regulatory network of the STRIPAK complex and the Hippo (SIN/MEN-MOR/RAM) pathways. Dettman et al. [85] identified an interaction between STK-24 (STK-6, MST-1), the *N*. *crassa* homolog of SmKIN24, and components of the MAK-2 mitogen activated protein kinases module, (MAPKKK) NRC-1 and (MAPKK) MEK-2, involved in fungal communication between germinating spores [86, 87]. All three kinases of the MAK-2 MAPK module were shown to be weakly associated with STRIPAK components PP2A-A, PP2A-C, striatin and MOB-3 in *N*. *crassa* [85]. Thus, STRIPAK components and the SmKIN24 homolog function together in at least one cascade in *N*. *crassa*. In mammals, CCM3 recruits the GCKs MST4, STK24 and STK25 but not MINK1 to striatin [15, 17, 45]. However, a homolog of CCM3 has so far not been identified in fungi. Thus, we assume that STRIPAK-associated kinases in *S*. *macrospora* are not recruited by other proteins to PRO11.

### SmKIN3 and SmKIN24 affect growth rate, sexual development and septum formation

Deletion of *Smkin3* does not impair fertility, but leads to an increased formation of aerial hyphae and reduced growth rate. In contrast, the deletion strain ΔSmkin24 was sterile, its lifecycle was halted at the later stage of protoperithecia formation and vegetative growth was also impaired (Fig 6). A global phenotypic analysis of serine/threonine-protein kinase deletion mutants in *N*. *crassa* revealed that the *N*. *crassa Smkin3*-homolog *stk-3* (*prk-9*, *sid-1*) affects asexual and sexual development as well as vegetative growth and aerial hyphae formation [88]. *S*. *macrospora* undergoes only sexual development, so we cannot comment on asexual reproduction in this study. The reduced growth rate of deletion strain ΔSmkin3 confirmed previous results of Park *et al*. [88], however, in contrast to these findings, deletion of *Smkin3* did not impair fruiting-body development or fertility. Heilig *et al*. [78] reported that *N*. *crassa* wt x Δstk-3 (prk-9, sid-1) crosses exhibited no abnormalities during sexual development. Deletion of *stk-24* (*stk-6*, *mst-1*) in *N*. *crassa* resulted in reduced aerial hyphae and slightly reduced macroconidia production [89]. Similar to the *S*. *macrospora* ΔSmkin24 mutant which is halted at later stages of protoperithecia formation, *N*. *crassa* Δstk-24 (stk-6, mst-1) x Δstk-24 (stk-6, mst-1) crosses resulted in empty perithecia that do not contain ascospores. Unlike *S*. *macrospora* mutant ΔSmkin24, the mycelial extension rates were not impaired in the *N*. *crassa* Δstk-24 (stk-6, mst-1) mutant [35]. Both *N*. *crassa* Δstk-3 (prk-9, sid-1) and Δstk-24 (stk-6, mst-1) mutants showed hyphal tip swelling and bursting of hyphal tips followed by cytoplasmic leakage [78, 89]. This phenotype was not observed in the *S*. *macrospora* ΔSmkin3 and ΔSmkin24 mutants. Deletion of *Smkin3* and *Smkin24* cause defects in septation, and the SmKIN3 and SmKIN24 proteins are localized to the septa (Fig 7). In *N*. *crassa*, the respective homologs are also localized to the septum and deletion of the genes causes defects in septa formation [35, 78, 89].

Mutant ΔSmkin3 exhibited fewer septa compared with the wt strain, and hyphal compartments were therefore elongated (Fig 8). This phenotype reverted, as growth continued beyond 24 h, presumably due to the accumulation of suppressor mutations. This is similar to the *N*. *crassa* Δstk-3 (prk-9, sid-1) mutant that produces initially aseptate germlings that form septa during the later stages of colony development [78]. In addition, germlings derived from ΔSmkin3 protoplasts grew faster than germlings of wt protoplasts (Fig 9). The septation phenotype of mutants ΔSmkin24 and ΔSmkin3 were distinct, mutant ΔSmkin24 generated more septa that aggregated into bundle-like structures of abnormal shape (Fig 8). Septal actomyosin tangle (SAT) assembly, cortical actomyosin ring (CAR) assembly and CAR constriction are three consecutive stages of septum formation in *N*. *crassa* [90]. The septa observed in mutant ΔSmkin24 resembled unfinished septa from the early stages of septum formation, that were observed during MYO-2 GFP localization studies of SAT and CAR assembly in *N*. *crassa* [90]. SmKIN24 is therefore required for proper CAR assembly, as was recently shown by Heilig *et al*. [35] in *N*. *crassa*. Despite their contrary phenotypes, SmKIN3 and SmKIN24 appear to function independently in *S*. *macrospora*, as shown by the double-deletion mutant ΔSmkin3/ΔSmkin24 that exhibited a phenotype that combined the phenotypes of both single-deletion strains, although septum formation resembles that of the ΔSmkin24 mutant (Fig 8).

The *N*. *crassa* homologs of SmKIN3 and SmKIN24 are implicated in SIN and MOR pathways as discussed above. The results of the present study indicate an additional role of these GCKs in *S*. *macrospora*, potentially via interaction with the striatin homolog PRO11. The interaction between PRO11 and SmKIN3 or SmKIN24 reported here for the first time, should be confirmed by other *in vivo* methods, such as bimolecular fluorescence complementation coupled with high resolution microscopy, which could also provide more detailed localization information of a possible interaction. In addition, interactome studies could identify transient interaction partners and may give insights into crosstalk between signaling pathways and modulation by protein complexes.

## Supporting Information

S1 File **Table A**, Plasmids used for this study. **Table B**, Primers used for this study. **Fig A**, RT-PCR analysis of *Smkin3* and *Smkin24*. (A) Schematic illustration of the genes *Smkin3* and *Smkin24*. Introns are indicated as grey boxes, positions of primers used for analysis of intron splicing are indicated by arrows. (B) Results of the RT-PCR analysis. Shown are the obtained amplicons for respective primer pairs from cDNA and gDNA. (C) Schematic illustration of *Smkin3* and *Smkin24* transcripts identified by cDNA sequencing. **Fig B**, Alignment of aa sequences encoded by alternatively spliced *Smkin24* transcripts. *Smkin24* without introns results in a protein of 882 aa and thus representing the largest protein. Expression of *Smkin24* with remaining intron I but removed intron II-IV results in a protein comprising aa 94–882 of the protein derived from *Smkin24* without introns. *Smkin24* with spliced intron I-III but remaining intron IV results in a protein comprising aa 1–729. **Fig C**, Identity of aligned amino-acid sequences in pair-wise comparison. **Fig D**, Controls of yeast two-hybrid assay AD-PRO11 and AD-SmMOB3 with empty vector pGBKT7. Shown are serial dilutions of diploid yeast strains obtained after mating and spread on SD medium lacking tryptophan (trp) and leucine (leu) or trp, leu and adenine (ade) to test the interaction of both proteins. The left picture displays a control, which ensures presence of both plasmids in the diploid strain; trp and leu prototrophy are regained by genes present on the plasmid. The right picture displays the interaction assay; ade prototrophy is not obtained by negative interaction between the GAL4 binding domain (BD) and proteins PRO11 and SmMOB3 fused GAL4 activation domain (AD). **Fig E**, Co-IP of FLAG-SmKIN3 and HA-PRO11 without crosslinker added. As a positive control a cell extract of a strain expressing the respective protein in suitable amounts (cell extract) is shown in the left lane; to exclude unspecific antibody binding, cell extracts of the wt was used as second negative control for Western blot analysis (right lane). The co-IP without crosslinker added are shown in the middle lane. (A) FLAG-SmKIN3 detected by FLAG antibody (anti-FLAG) (B) HA-PRO11 by anti-HA antibody (anti-HA). **Fig F**, Generation of the ΔSmkin3 deletion strain. (A) Schematic illustration of the *Smkin3* locus before and after homologous integration of the deletion cassette. Primers used for verification of the deletion strain are indicated by arrows. Sizes of PCR fragments and the probe used for Southern hybridization are given. (B) Verification of the respective deletion using PCR. Sizes of amplicons and positions of the primers as indicated in (A). (C) Integration of the deletion cassette was verified by Southern hybridization [46]. Positions of the respective probes are indicated in (A). ΔSmkin3 was verified using a hygromycin specific probe that only binds within the deletion cassette. **Fig G**, Generation of the ΔSmkin24 deletion strain. (A) Schematic illustration of the *Smkin24* locus before and after homologous integration of the deletion cassette. Primers used for verification of the deletion strain are indicated by arrows. Sizes of PCR fragments and the probe used for Southern hybridization are given. (B) Verification of the respective deletion using PCR. Sizes of amplicons and positions of the primers as indicated in (A). (**C**) Integration of the deletion cassette was verified by Southern hybridization [46]. Positions of the respective probes are indicated in (A). ΔSmkin24 was verified using a probe binding at the 3’ region of *Smkin24*. The successful integration is represented by a band shift. **Fig H**, Macroscopic and microscopic analysis of the sexual development of wt, complemented ΔSmkin3 (ΔSmkin3+), complemented ΔSmkin24 (ΔSmkin24+) and partially complemented ΔSmkin3/ΔSmkin24. The wt strain produces ascogonia after 3 days which develop to unpigmented protoperithecia at day 4 and pigmented protoperithecia at day 5. After 7 days mature perithecia with asci and ascospores are formed. Similar to the wt ΔSmkin3+ and ΔSmkin24+ and ΔSmkin3/ΔSmkin24 + *Smkin24* completed the lifecycle within 7 days and produced mature ascospores. Development of ΔSmkin3/ΔSmkin24 + *Smkin3* is arrested at stage of late protoperithecia formation, similar to ΔSmkin24. Scale bars as indicated. **Fig I**, Microscopic investigation of hyphal fusion in wt, ΔSmkin3, ΔSmkin24 and ΔSmkin3/ΔSmkin24. ΔSmkin3, ΔSmkin24 and ΔSmkin3/ΔSmkin24 are capable of hyphal fusion. Hyphal fusion events are highlighted with circles. Pictures of hyphal fusion events were taken at subperiphal regions 10 mm behind the growth front. Hyphal fusion was investigated 2–3 days past inoculation.
(PDF)
