# Supplementary material for: Germinal Center Kinases SmKIN3 and SmKIN24 Are Associated with the Sordaria macrospora Striatin-Interacting Phosphatase and Kinase (STRIPAK) Complex
Source: PLoS One. 2015 Sep 29;10(9):e0139163. doi: 10.1371/journal.pone.0139163 (PMC4587736; doi:10.1371/journal.pone.0139163)
Supplement: S1 File — Table A, Plasmids used for this study. Table B, Primers used for this study. Fig A, RT-PCR analysis of Smkin3 and Smkin24. (A) Schematic illustration of the genes Smkin3 and Smkin24. Introns are indicated as grey boxes, positions of primers used for analysis of intron splicing are indicated by arrows. (B) Results of the RT-PCR analysis. Shown are the obtained amplicons for respective primer pairs from cDNA and gDNA. (C) Schematic illustration of Smkin3 and Smkin24 transcripts identified by cDNA sequencing. Fig B, Alignment of aa sequences encoded by alternatively spliced Smkin24 transcripts. Smkin24 without introns results in a protein of 882 aa and thus representing the largest protein. Expression of Smkin24 with remaining intron I but removed intron II-IV results in a protein comprising aa 94–882 of the protein derived from Smkin24 without introns. Smkin24 with spliced intron I-III but remaining intron IV results in a protein comprising aa 1–729. Fig C, Identity of aligned amino-acid sequences in pair-wise comparison. Fig D, Controls of yeast two-hybrid assay AD-PRO11 and AD-SmMOB3 with empty vector pGBKT7. Shown are serial dilutions of diploid yeast strains obtained after mating and spread on SD medium lacking tryptophan (trp) and leucine (leu) or trp, leu and adenine (ade) to test the interaction of both proteins. The left picture displays a control, which ensures presence of both plasmids in the diploid strain; trp and leu prototrophy are regained by genes present on the plasmid. The right picture displays the interaction assay; ade prototrophy is not obtained by negative interaction between the GAL4 binding domain (BD) and proteins PRO11 and SmMOB3 fused GAL4 activation domain (AD). Fig E, Co-IP of FLAG-SmKIN3 and HA-PRO11 without crosslinker added. As a positive control a cell extract of a strain expressing the respective protein in suitable amounts (cell extract) is shown in the left lane; to exclude unspecific antibody binding, cell extracts of the wt was u [file pone.0139163.s001.pdf]

S1 Supporting Information

Germinal Center Kinases SmKIN3 and SmKIN24 are associated with the *Sordaria macrospora* striatin-interacting phosphatase and kinase (STRIPAK) complex

Stefan Frey[1], Eva J. Reschka[1], Stefanie Pöggeler[1,2]

S1File. Supporting information Plasmids used for this study **(Table A.)**. .Primers used for this study **(Table B)**. RT-PCR analysis of *Smkin3* and *Smkin24*. (A) Schematic illustration of the genes *Smkin3* and *Smkin24*. Introns are indicated as grey boxes, positions of primers used for analysis of intron splicing are indicated by arrows. (B) Results of the RT-PCR analysis. Shown are the obtained amplicons for respective primer pairs from cDNA and gDNA. (C) Schematic illustration of *Smkin3* and *Smkin24* transcripts identified by cDNA sequencing (**Figure A**). Alignment of aa sequences encoded by alternatively spliced *Smkin24* transcripts. *Smkin24* without introns results in a protein of 882 aa and thus representing the largest protein. Expression of *Smkin24* with remaining intron I but removed intron II-IV results in a protein comprising aa 94-882 of the protein derived from *Smkin24* without introns. *Smkin24* with spliced intron I-III but remaining intron IV results in a protein comprising aa 1-729 (**Figure B**). Identity of aligned amino-acid sequences in pair-wise comparison **(Figure C).** Controls of yeast two-hybrid assay AD-PRO11 and AD-SmMOB3 with empty vector pGBKT7. Shown are serial dilutions of diploid yeast strains obtained after mating and spread on SD medium lacking tryptophan (trp) and leucine (leu) or trp, leu and adenine (ade) to test the interaction of both proteins. The left picture displays a control, which ensures presence of both plasmids in the diploid strain; trp and leu prototrophy are regained by genes present on the plasmid. The right picture displays the

interaction assay; ade prototrophy is not obtained by negative interaction between the GAL4 binding domain (BD) and proteins PRO11 and SmMOB3 fused GAL4 activation domain (AD) (**FigureD**). Co-IP of FLAG-SmKIN3 and HA-PRO11 without crosslinker added. As a positive control a cell extract of a strain expressing the respective protein in suitable amounts (cell extract) is shown in the left lane; to exclude unspecific antibody binding, cell extracts of the wt was used as second negative control for Western blot analysis (right lane). The co-IP without crosslinker added are shown in the middle lane. (A) FLAG-SmKIN3 detected by FLAG antibody (anti-FLAG) (B) HA-PRO11 by anti-HA antibody (anti-HA) (**Figure E**). Generation of the ΔSmkin3 deletion strain. (A) Schematic illustration of the *Smkin3* locus before and after homologous integration of the deletion cassette. Primers used for verification of the deletion strain are indicated by arrows. Sizes of PCR fragments and the probe used for Southern hybridization are given. (B) Verification of the respective deletion using PCR. Sizes of amplicons and positions of the primers as indicated in (A). (C) Integration of the deletion cassette was verified by Southern hybridization [46]. Positions of the respective probes are indicated in (A). ΔSmkin3 was verified using a hygromycin specific probe that only binds within the deletion cassette (**Figure F**). Generation of the ΔSmkin24 deletion strain. (A) Schematic illustration of the *Smkin24* locus before and after homologous integration of the deletion cassette. Primers used for verification of the deletion strain are indicated by arrows. Sizes of PCR fragments and the probe used for Southern hybridization are given. (**B**) Verification of the respective deletion using PCR. Sizes of amplicons and positions of the primers as indicated in (A). (**C**) Integration of the deletion cassette was verified by Southern hybridization [46]. Positions of the respective probes are indicated in (A). ΔSmkin24 was verified using a probe binding at the 3' region of *Smkin24*. The successful integration is represented by a band shift

(**Figure G**). Macroscopic and microscopic analysis of the sexual development of wt, complemented ΔSmkin3 (ΔSmkin3+), complemented ΔSmkin24 (ΔSmkin24+) and partially complemented ΔSmkin3/ΔSmkin24. The wt strain produces ascogonia after 3 days which develop to unpigmented protoperithecia at day 4 and pigmented protoperithecia at day 5. After 7 days mature perithecia with asci and ascospores are formed. Similar to the wt ΔSmkin3+ and ΔSmkin24+ and ΔSmkin3/ΔSmkin24 + *Smkin24* completed the lifecycle within 7 days and produced mature ascospores. Development of ΔSmkin3/ΔSmkin24 + *Smkin3* is arrested at stage of late protoperithecia formation, similar to ΔSmkin24. Scale bars as indicated (**Figure H**). Microscopic investigation of hyphal fusion in wt, ΔSmkin3, ΔSmkin24 and ΔSmkin3/ΔSmkin24. ΔSmkin3, ΔSmkin24 and ΔSmkin3/ΔSmkin24 are capable of hyphal fusion. Hyphal fusion events are highlighted with circles. Pictures of hyphal fusion events were taken at subperiphal regions 10 mm behind the growth front. Hyphal fusion was investigated 2-3 days past inoculation (**Figure I**).

Tables and Figure legends

Table A. Plasmids used for this study

Name	Features	Reference
pDS23-egfp	*egfp* under control of the *gpd* promoter and *trpC* terminator of *Aspergillus nidulans*, *URA3, nat*-cassette	[1]
pRSnat	*URA3, nat* cassette, amp^R	[2]
pRShyg	*URA3, hph* cassette, amp^R	[3]

pFLAGN1	*his-3::ccg-1(p)::3xFLAG*	[4]
pHAN1	*his-3::ccg-1(p)::HA*	[4]
pRS-SmKIN3+	*Smkin3* bp -1038 to 3811 in pRSnat	this study
pDS-SmKIN3ngfp	*Smkin3* bp 1 to 2755 in pDS23, *egfp* is fused upstream to *Smkin3*	this study
pRS-Smkin+	*Smkin24* bp -1036 to 4023 in pRSnat	this study
pDS-SmKIN24ngfp	*Smkin24* bp 1 to 2947 in pDS23, *egfp* is fused upstream to *Smkin24*	this study
pRS-KoSmkin3	1038 bp of the upstream region and 1016 bp of the downstream region of *Smkin3* interrupted by the *hph*-cassette in pRSnat	this study
pRS-KoSmkin24	1036 bp of the upstream region and 756 bp of the downstream region of *Smkin24* interrupted by the *hph*-cassette in pRSnat	this study
pGADT7	Yeast two-hybrid vector with	Clonetech Laboratories Inc.

	GAL4 activation domain	(Saint Germain-en Laye, France, 630442)
pGBKT7	Yeast two-hybrid vector with GAL4 DNA binding domain domain	Clonetech Laboratories Inc. (Saint Germain-en Laye, France, 630489)
pAD-RanBPM	RanBPM fused to GAL4 AD	[5]
pAD-SmMOB3	*Smmob3* cDNA bp 1 to 1992 fused to GAL4 AD in pGADT7	[3]
pAD11FL	*pro11 full-length* cDNA fused to GAL4 AD in pGADT7	[3]
pBD-SmKIN3	*Smkin3* cDNA bp 1-2463 fused to GAL4 BD in pGBKT7	this study
pBD-SmKIN24	*Smkin24* cDNA bp 1-2616 fused to GAL4 BD in pGBKT7	this study
pFLAG-SmKIN3	*ccg1(p)::3xFLAG::Smkin3* in pRShyg under control of the *N. crassa ccg1* promoter and *trpC* terminator of *Aspergillus nidulans*	this study
pHA11	*ccg1(p)::HA::pro11* in pRSnat	[3]

Table B. Primers used for this study

Name	Sequence
Smkin3_1k_5F	GTAACGCCAGGGTTTTCCCAGTCACGACGCGACTCGACAGGCATGCGAA
Smkin3_5R	CAAAAAATGCTCCTTCAATATCAGTTAACCTTTTGGTTACAGAAGGGTG
Smkin3_3F	GAGTAGATGCCGACCGGGAACCAGTTAACTAGTGAGGTGATGAATGGTG
Smkin3_1k_3R	GCGGATAACAATTTCACACAGGAAACAGCATCGCTTCATGACTCCCCGG
Smkin3_5F	GACTGCCCCGGCGCGGCAGC
Smkin3_3R	CAACGTAGGTATGTACGTAG
Smkin24_1k_5F	GTAACGCCAGGGTTTTCCCAGTCACGACGCAGTGAGCTAAGTGCTAACC
Smkin24_5R	GTAACGCCAGGGTTTTCCCAGTCACGACGCGATTAAGGAGGCTGGCCTG
Smkin24_3F	GAGTAGATGCCGACCGGGAACCAGTTAACTAGTTAGAGGACTTGCATAT
Smkin24_1k_3R	GCGGATAACAATTTCACACAGGAAACAGCGACAGTGTAAGGGTACCTAC
Smkin24_2k_3R	ACTTTGATGGAAGGCTTGGTG
Smkin24_5F	GACATGCCTGCCCCACAAAT

Smkin24_F	ATGGCCGACCGCGAATATGA
Smkin3_F	ATGGCCGACGAAGGAGTCGC
Smkin3_R	CTAAGATCCGGCAACAGCCC
3int 1-3_R	TGCTTAATGACCTCGGGAGCCA
24int 1-3_R	TGCTTAATGACCTCGGGAGCCA
24int 4_F	CCTTCGATGCTCTATCACCAGC
24int 4_R	CCAGCTTATACACCAACTTGCGTATC
Smkin3ngfp_F	TCACTCTCGGCATGGACGAGCTGTACAAGATGGCCGACGAAGGAGTCGC
Smkin3ngfp_R	GTTTGATGATTTCAGTAACGTTAAGTGGATCATCTGTTCACCTTCTCTT
Smkin24ngfp_F	TCACTCTCGGCATGGACGAGCTGTACAAGATGGCCGACCGCGAATATGA
Smkin24ngfp_R	GTTTGATGATTTCAGTAACGTTAAGTGGATCATGTTCCTTGTTTCATTC
kin3_pBD_inf_F	AGGAGGACCTGCATATGGCCGACGAAGGAGTCGC
kin3_pBD_inf_R	GGATCCCCGGGAATTCTCATCTGTTCACCTTCTCTT
kin24_pBD_inf_F	AGGAGGACCTGCATATGGCCGACCGCGAATATGA
kin24_pBD_inf_R	GGATCCCCGGGAATTCTCATGTTCCTTGTTTCATTCCC
kin3_FLAG_F	ATTACAAGGATGACGATGACAAGGGTTCAATGGCCGACGAAGGAGTCGC
kin3_TtrpC_R	GTTTGATGATTTCAGTAACGTTAAGTGGATCATCTGTTCACCTTCTCTT

TtrpC_F	TCCACTTAACGTTACTGAAAT
pRS426GFPrev	GCGGATAACAATTTCACACAGGAAACAGCTCGAGTGGAGATGT GGAGTG
pRSccg1	GTAACGCCAGGGTTTTCCCAGTCACGACG TAGAAGGAGCAGTCCA

Figure A. RT-PCR analysis of *Smkin3* and *Smkin24*. (A) Schematic illustration of the genes *Smkin3* and *Smkin24*. Introns are indicated as grey boxes, positions of primers used for analysis of intron splicing are indicated by arrows. (**B**) Results of the RT-PCR analysis. Shown are the obtained amplicons for respective primer pairs from cDNA and gDNA. **(C)** Schematic illustration of *Smkin3* and *Smkin24* transcripts identified by cDNA sequencing.

Figure B. Alignment of aa sequences encoded by alternatively spliced *Smkin24* transcripts. *Smkin24* without introns results in a protein of 882 aa and thus representing the largest protein. Expression of *Smkin24* with remaining intron I but removed intron II-IV results in a protein comprising aa 94-882 of the protein derived from *Smkin24* without introns. *Smkin24* with spliced intron I-III but remaining intron IV results in a protein comprising aa 1-729.

Figure C. **Identity of aligned amino-acid sequences in pair-wise comparison.**

Figure D. Negative controls of yeast two-hybrid assay AD-PRO11 and AD-SmMOB3 with empty vector pGBKT7. Shown are serial dilutions of diploid yeast strains obtained after mating

and spread on SD medium lacking tryptophan (trp) and leucine (leu) or trp, leu and adenine (ade) to test the interaction of both proteins. The left picture displays a control, which ensures presence of both plasmids in the diploid strain; trp and leu prototrophy are regained by genes present on the plasmid. The right picture displays the interaction assay; ade prototrophy is not obtained by negative interaction between the GAL4 binding domain (BD) and proteins PRO11 and SmMOB3 fused GAL4 activation domain (AD).

Figure E. **Co-IP of FLAG-SmKin3 and HA-PRO11 without crosslinker added.** As a positive control a cell extract of a strain expressing the respective protein in suitable amounts (cell extract) is shown in the left lane; to exclude unspecific antibody binding, cell extracts of the wt was used as second negative control for Western blot analysis (right lane). The co-IP without crosslinker added are shown in the middle lane. **(A)** FLAG-SmKIN3 detected by FLAG antibody (anti-FLAG) **(B)** HA-PRO11 by anti-HA antibody (anti-HA).

Figure F. Generation of the ΔSmkin3 deletion strain. (**A**) Schematic illustration of the *Smkin3* locus before and after homologous integration of the deletion cassette. Primers used for verification of the deletion strain are indicated by arrows. Sizes of PCR fragments and the probe used for Southern hybridization are given. (**B**) Verification of the respective deletion using PCR. Sizes of amplicons and positions of the primers as indicated in (A). (**C**) Integration of the deletion cassette was verified by Southern hybridization (6). Positions of the respective probes are indicated in (A). ΔSmkin3 was verified using a hygromycin specific probe that only binds within the deletion cassette.

Figure G. Generation of the ΔSmkin24 deletion strain. (**A**) Schematic illustration of the *Smkin24* locus before and after homologous integration of the deletion cassette. Primers used for verification of the deletion strain are indicated by arrows. Sizes of PCR fragments and the probe used for Southern hybridization are given. (**B**) Verification of the respective deletion using PCR. Sizes of amplicons and positions of the primers as indicated in (A). (**C**) Integration of the deletion cassette was verified by Southern hybridization [6]. Positions of the respective probes are indicated in (A). ΔSmkin24 was verified using a probe binding at the 3' region of *Smkin24*. The successful integration is represented by a band shift.

Figure H. Macroscopic and microscopic analysis of the sexual development of wt, complemented ΔSmkin3 (ΔSmkin3+), complemented ΔSmkin24 (ΔSmkin24+) and partially complemented ΔSmkin3/ΔSmkin24. The wt strain produces ascogonia after 3 days which develop to unpigmented protoperithecia at day 4 and pigmented protoperithecia at day 5. After 7 days mature perithecia with asci and ascospores are formed. Similar to the wt ΔSmkin3+ and ΔSmkin24+ and ΔSmkin3/ΔSmkin24 + *Smkin24* completed the lifecycle within 7 days and produced mature ascospores. Development of ΔSmkin3/ΔSmkin24 + *Smkin3* is arrested at stage of late protoperithecia formation, similar to ΔSmkin24. Scale bars as indicated.

Figure I. Microscopic investigation of hyphal fusion in wt, ΔSmkin3, ΔSmkin24 and ΔSmkin3/ΔSmkin24. ΔSmkin3, ΔSmkin24 and ΔSmkin3/ΔSmkin24 are capable of hyphal fusion. Hyphal fusion events are highlighted with circles. Pictures of hyphal fusion events were taken at subperiphal regions 10 mm behind the growth front. Hyphal fusion was investigated 2-3 days past inoculation.

References

1. Teichert I, Wolff G, Kück U, Nowrousian M. Combining laser microdissection and RNA-seq to chart the transcriptional landscape of fungal development. BMC genomics. 2012;13:511. Epub 2012/09/29.
2. Klix V, Nowrousian M, Ringelberg C, Loros JJ, Dunlap JC, Pöggeler S. Functional characterization of MAT1-1-specific mating-type genes in the homothallic ascomycete *Sordaria macrospora* provides new insights into essential and nonessential sexual regulators. Eukaryot Cell. 2010;9(6):894-905. Epub 2010/05/04.
3. Bloemendal S, Bernhards Y, Bartho K, Dettmann A, Voigt O, Teichert I, et al. A homologue of the human STRIPAK complex controls sexual development in fungi. Molecular microbiology. 2012;84(2):310-23. Epub 2012/03/02.
4. Kawabata T, Inoue H. Detection of physical interactions by immunoprecipitation of FLAG- and HA-tagged proteins expressed at the *his-3* locus in *Neurospora crassa*. Fungal Genetics Newsletter. 2007.
5. Tucker CL, Peteya LA, Pittman AM, Zhong J. A genetic test for yeast two-hybrid bait competency using RanBPM. Genetics. 2009;182(4):1377-9. Epub 2009/06/03.
6. Sambrook J, Fritsch E, Maniatis T. Molecular cloning: a laboratory manual. 2nd ed. Cold Spring Harbor: Cold Spring Harbor Laboratory Press; 2001.

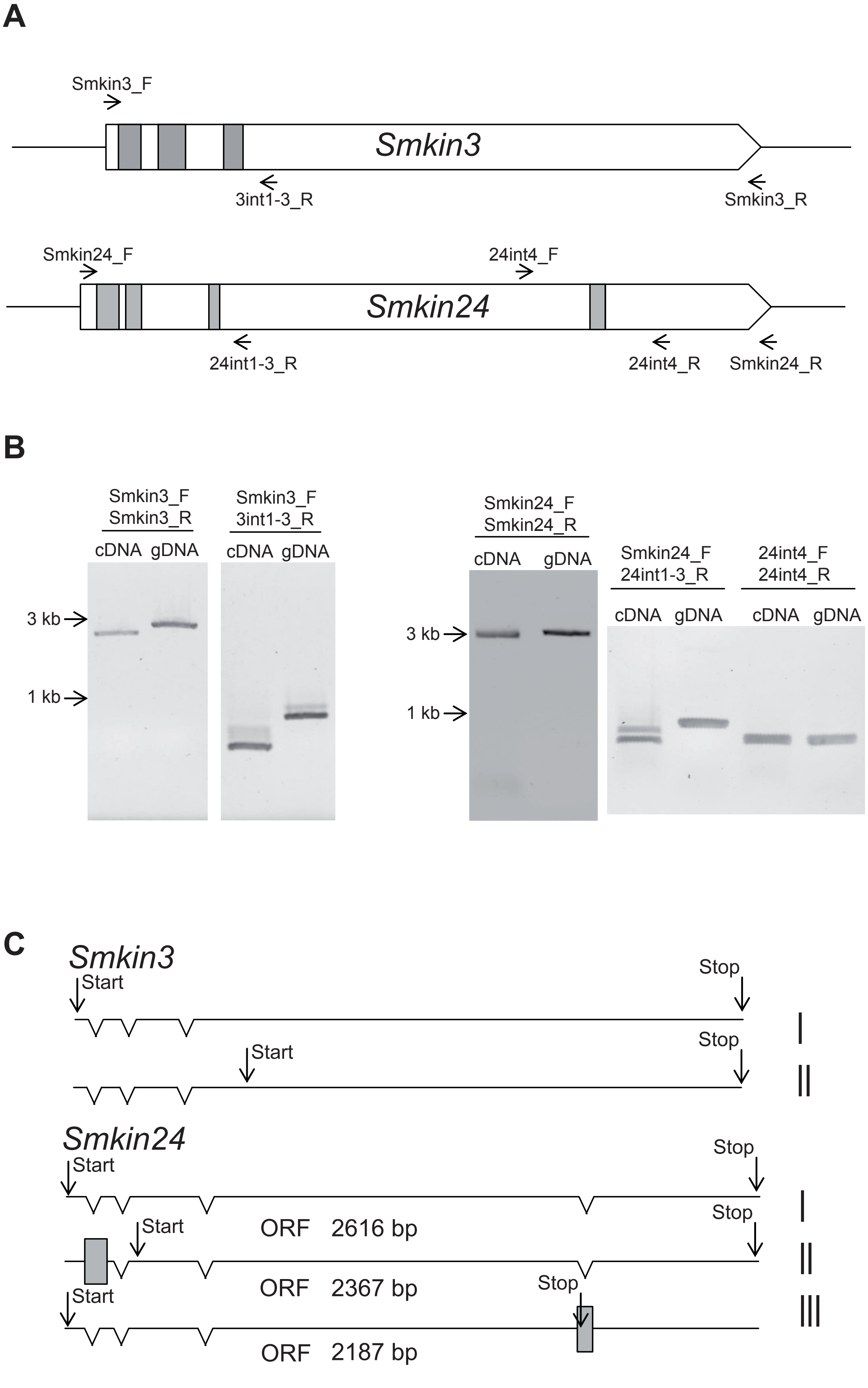
A
Smkin3_F
Smkin3
3int1-3_R
Smkin3_R
Smkin24_F
24int4_F
Smkin24
24int1-3_R
24int4_R
Smkin24_R
B
Smkin3_F
Smkin3_R
cDNA gDNA
Smkin3_F
3int1-3_R
cDNA gDNA
3 kb
1 kb
Smkin24_F
Smkin24_R
cDNA gDNA
Smkin24_F
24int1-3_R
24int4_F
24int4_R
cDNA gDNA
cDNA gDNA
3 kb
1 kb
C
Smkin3
Start
Stop
I
Start
Stop
II
Smkin24
Start
Stop
I
Start
ORF 2616 bp
Stop
II
ORF 2367 bp
Start
Stop
III
ORF 2187 bp

S1, Fig. A

SmKIN24

```
I   MADREYDEHEGSVDPELLYTKEFCIGGGSFGKVYKGVDKRSGQAVAIKVIDIESAEDEVEDIIQEIAILSELQSPYVTKYYGSYAKGAELWIVMEFCSGGSCADLMKP
II  ---------------------------------------------------------------------------------------------MEFCSGGSCADLMKP
III MADREYDEHEGSVDPELLYTKEFCIGGGSFGKVYKGVDKRSGQAVAIKVIDIESAEDEVEDIIQEIAILSELQSPYVTKYYGSYAKGAELWIVMEFCSGGSCADLMKP

    GLIGEDYIAIIVRELLLGLDYLHQDKKLHRDIKAANILLAANGQVKLADFGVSGQLSATMTKKNTFVGTPFWMAPEVIKQSGYDHKADIWSLGITALELAKGEPPYAD
    GLIGEDYIAIIVRELLLGLDYLHQDKKLHRDIKAANILLAANGQVKLADFGVSGQLSATMTKKNTFVGTPFWMAPEVIKQSGYDHKADIWSLGITALELAKGEPPYAD
    GLIGEDYIAIIVRELLLGLDYLHQDKKLHRDIKAANILLAANGQVKLADFGVSGQLSATMTKKNTFVGTPFWMAPEVIKQSGYDHKADIWSLGITALELAKGEPPYAD

    IHPMKVLFLIPKNPPPRLEGNFSKGFKEFIELCLQRDPKERPTARELLKHPFIRRAKKTSYLTELIERYNRWAATHKQDDEEYNEPEQNHYVEQRKHINEDMWDFGTV
    IHPMKVLFLIPKNPPPRLEGNFSKGFKEFIELCLQRDPKERPTARELLKHPFIRRAKKTSYLTELIERYNRWAATHKQDDEEYNEPEQNHYVEQRKHINEDMWDFGTV
    IHPMKVLFLIPKNPPPRLEGNFSKGFKEFIELCLQRDPKERPTARELLKHPFIRRAKKTSYLTELIERYNRWAATHKQDDEEYNEPEQNHYVEQRKHINEDMWDFGTV

    RLVSDRGGVVHRPGMLNVMDESTLNARSGRANEIADNREDWRRESNQATSKLAVDTSGQGTARQASPARKPVTTPSLAMPPPTRVSVPSSPVKQQIQQLEQQSQKSRE
    RLVSDRGGVVHRPGMLNVMDESTLNARSGRANEIADNREDWRRESNQATSKLAVDTSGQGTARQASPARKPVTTPSLAMPPPTRVSVPSSPVKQQIQQLEQQSQKSRE
    RLVSDRGGVVHRPGMLNVMDESTLNARSGRANEIADNREDWRRESNQATSKLAVDTSGQGTARQASPARKPVTTPSLAMPPPTRVSVPSSPVKQQIQQLEQQSQKSRE

    TPTPRTSLARPVSHVPLGSSPDYDRVLQEQLRRDIGGMNLGPAIQNSTQQQSALSPPQYNQQPSSSSSRTSAGPMKLSEIPPFRGGQQPQQQPLQKLTNQQINQQAPS
    TPTPRTSLARPVSHVPLGSSPDYDRVLQEQLRRDIGGMNLGPAIQNSTQQQSALSPPQYNQQPSSSSSRTSAGPMKLSEIPPFRGGQQPQQQPLQKLTNQQINQQAPS
    TPTPRTSLARPVSHVPLGSSPDYDRVLQEQLRRDIGGMNLGPAIQNSTQQQSALSPPQYNQQPSSSSSRTSAGPMKLSEIPPFRGGQQPQQQPLQKLTNQQINQQAPS

    TNKPFQQQQQQQAPLYHQPSRESLYRQQQQQPPPPQQQRQLNHQASREQVYQQHQRQQSQPSMLYHQPSRESLYQQQQQQQHNNNNNNKHHQQQQQQQANNNNNNNKQ
    TNKPFQQQQQQQAPLYHQPSRESLYRQQQQQPPPPQQQRQLNHQASREQVYQQHQRQQSQPSMLYHQPSRESLYQQQQQQQHNNNNNNKHHQQQQQQQANNNNNNNKQ
    TNKPFQQQQQQQAPLYHQPSRESLYRQQQQQPPPPQQQRQLNHQASREQVYQQHQRQQSQPSMLYHQPSRESLYQQQQQQQHNNNNNNKHHQQQQQQQANNNNNNNKQ

    QQQQQQHWTATSLDNNNNNNKNNNNNNNNNTGLQPLLTTTTTTTKQPAAATAATAATTASATTASATKFTASAAAFLWTLPRSSPSGSRIVSTSSNYGGLSTPSSSSTS
    QQQQQQHWTATSLDNNNNNNKNNNNNNNNNTGLQPLLTTTTTTTKQPAAATAATAATTASATTASATKFTASAAAFLWTLPRSSPSGSRIVSTSSNYGGLSTPSSSSTS
    QQQQQQHWTATSLDNNNNNNKNNNNNNNNNTGLQPLLTTTTTTTKQPAAATAATAATTASATTASATKFTASAAAFLWTVST---------------------------

    LAAFPSPDPPSPSGELDALNDVIFPALEEALKRRQIMLQQTYRPEPGYAPSPPTPKQQRAEAANEKIRKLVYKLAHVCKEIDHYDKAEPVGMGKDVGGFLEGLLEEIL
    LAAFPSPDPPSPSGELDALNDVIFPALEEALKRRQIMLQQTYRPEPGYAPSPPTPKQQRAEAANEKIRKLVYKLAHVCKEIDHYDKAEPVGMGKDVGGFLEGLLEEIL
    ------------------------------------------------------------------------------------------------------------

    VRVEPLDVLGPEDGGRGG    aa 1-882
    VRVEPLDVLGPEDGGRGG    aa 94-882
    ------------------    aa1-729
```

S1, Fig. B

	MST4	STK24	STK25	MINK1	SmKIN24	NcSTK-6	FgESU11740	Pc21g04360	AfEDP48273	SmKIN3	NcPRK-9	AfEDP51073	Pc21g14960	Fg07344	SpSid1	ScKIC1	SpPpk11	ScSPS1
MST4	100 %	88 %	86 %	46 %	67 %	67 %	66%	66%	65 %	64 %	64 %	62 %	62 %	62 %	52 %	49 %	44 %	45 %
STK24		100 %	88 %	45 %	68 %	68 %	67 %	67 %	66 %	64 %	64 %	63 %	63 %	62 %	51 %	50 %	45 %	46 %
STK25			100 %	45 %	68 %	67 %	66 %	67 %	66 %	65 %	65 %	63 %	63 %	63 %	52 %	50 %	45 %	48 %
MINK1				100 %	47 %	46 %	47%	42 %	46 %	44 %	45 %	43 %	43 %	44 %	41 %	42 %	33%	37 %
SmKIN24					100 %	98 %	91 %	79 %	81 %	62 %	63 %	60 %	61 %	61 %	50 %	51 %	46 %	47 %
NcSTK-6						100 %	92 %	79 %	81 %	62 %	63 %	60 %	61 %	61 %	50 %	50 %	47 %	47 %
Fg ESU11740							100 %	78 %	82 %	63 %	65 %	62 %	62 %	63 %	50 %	49 %	46 %	47 %
Pc21g04360								100 %	84 %	60 %	61 %	59 %	59 %	58 %	52 %	48 %	44 %	47 %
Af EDP48273									100 %	60 %	62 %	58 %	81 %	58 %	51 %	48 %	45 %	49 %
SmKIN3										100 %	98 %	79 %	81 %	84 %	55 %	46 %	46 %	48 %
NcPRK-9											100 %	80 %	57 %	85 %	56 %	45 %	46 %	47 %
Af EDP51073												100 %	80 %	80 %	55 %	45 %	43 %	46 %
Pc21g14960													100 %	80 %	55 %	45 %	43 %	47 %
Fg07344														100 %	55 %	45 %	42 %	45 %
SpSid1															100 %	40 %	43 %	42 %
ScKIC1																100 %	35 %	42 %
SpPpk11																	100 %	44 %
ScSPS1																		100 %

S1, Fig. C

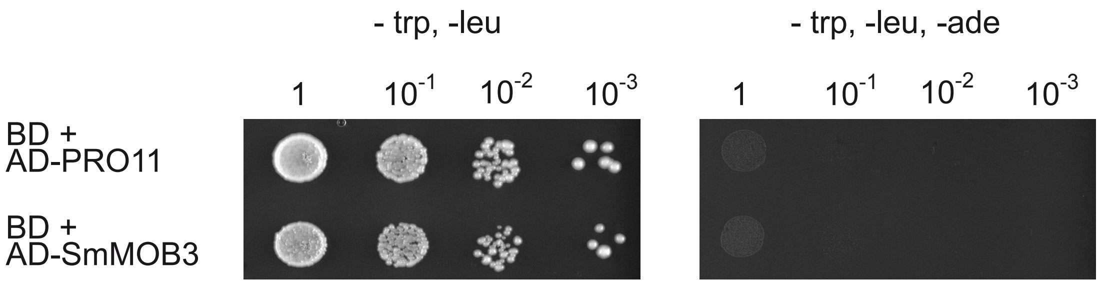
- trp, -leu
- trp, -leu, -ade
1
10-1
10-2
10-3
1
10-1
10-2
10-3
BD +
AD-PRO11
BD +
AD-SmMOB3

S1, Fig. D

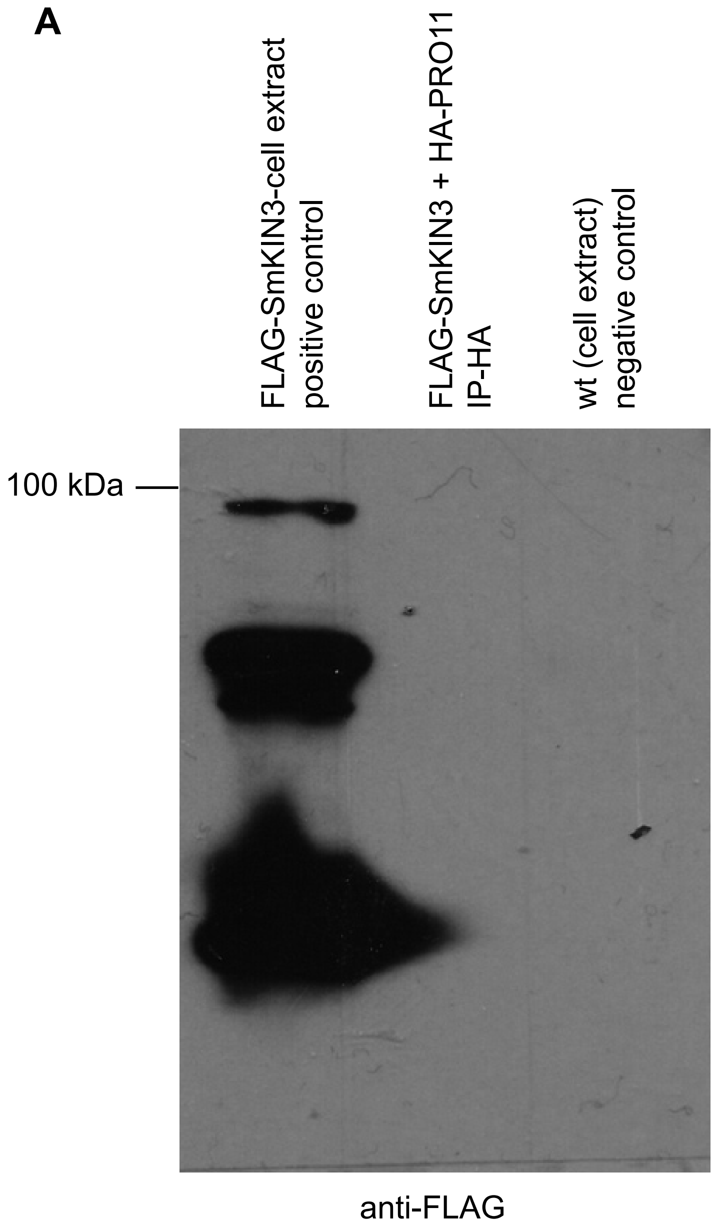
A
FLAG-SmKIN3-cell extract
positive control
FLAG-SmKIN3 + HA-PRO11
IP-HA
wt (cell extract)
negative control
100 kDa
anti-FLAG

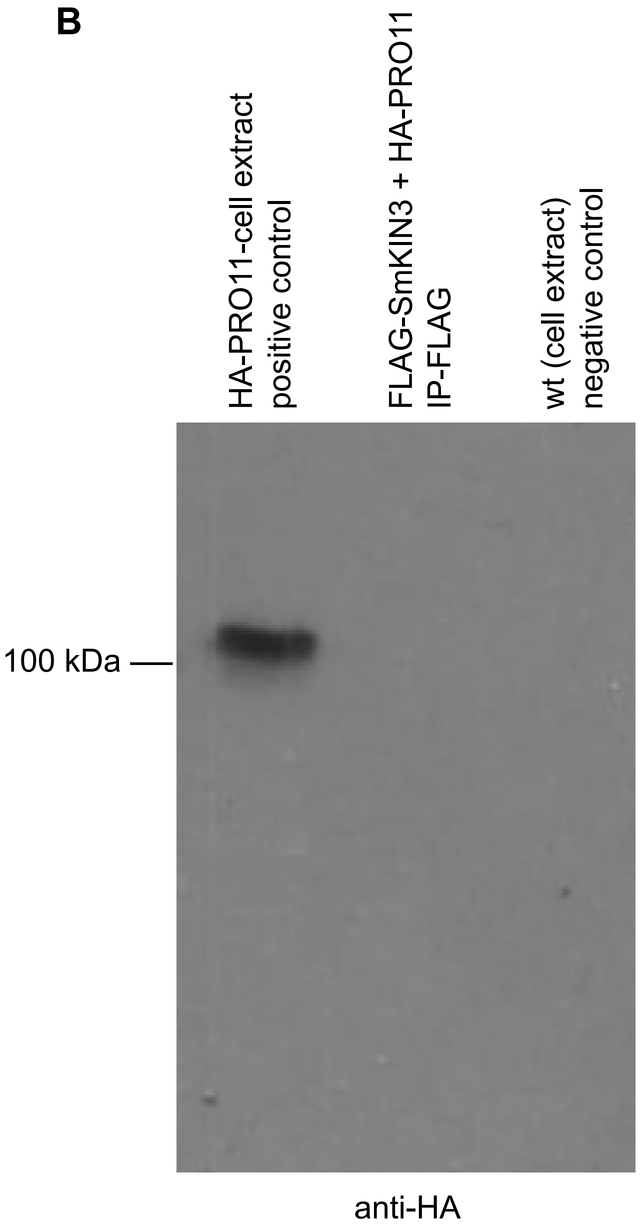
B
HA-PRO11-cell extract
positive control
FLAG-SmKIN3 + HA-PRO11
IP-FLAG
wt (cell extract)
negative control
100 kDa
anti-HA

S1, Fig. E

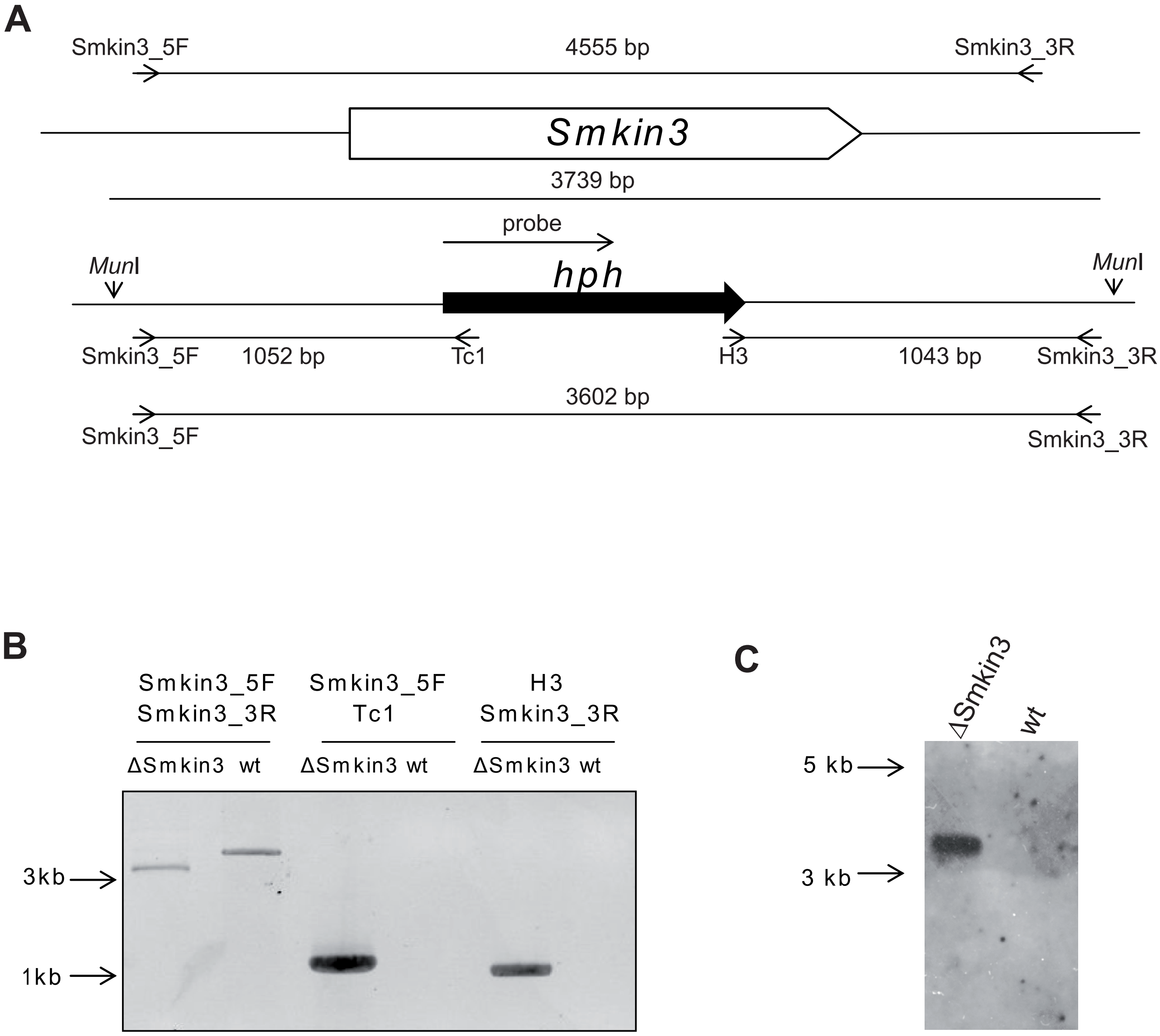
A
Smkin3_5F
4555 bp
Smkin3_3R
Smkin3
3739 bp
probe
MunI
hph
MunI
Smkin3_5F
1052 bp
Tc1
H3
1043 bp
Smkin3_3R
3602 bp
Smkin3_5F
Smkin3_3R
B
Smkin3_5F
Smkin3_3R
Smkin3_5F
Tc1
H3
Smkin3_3R
ΔSmkin3 wt
ΔSmkin3 wt
ΔSmkin3 wt
3kb
1kb
C
ΔSmkin3
wt
5 kb
3 kb

S1, Fig. F

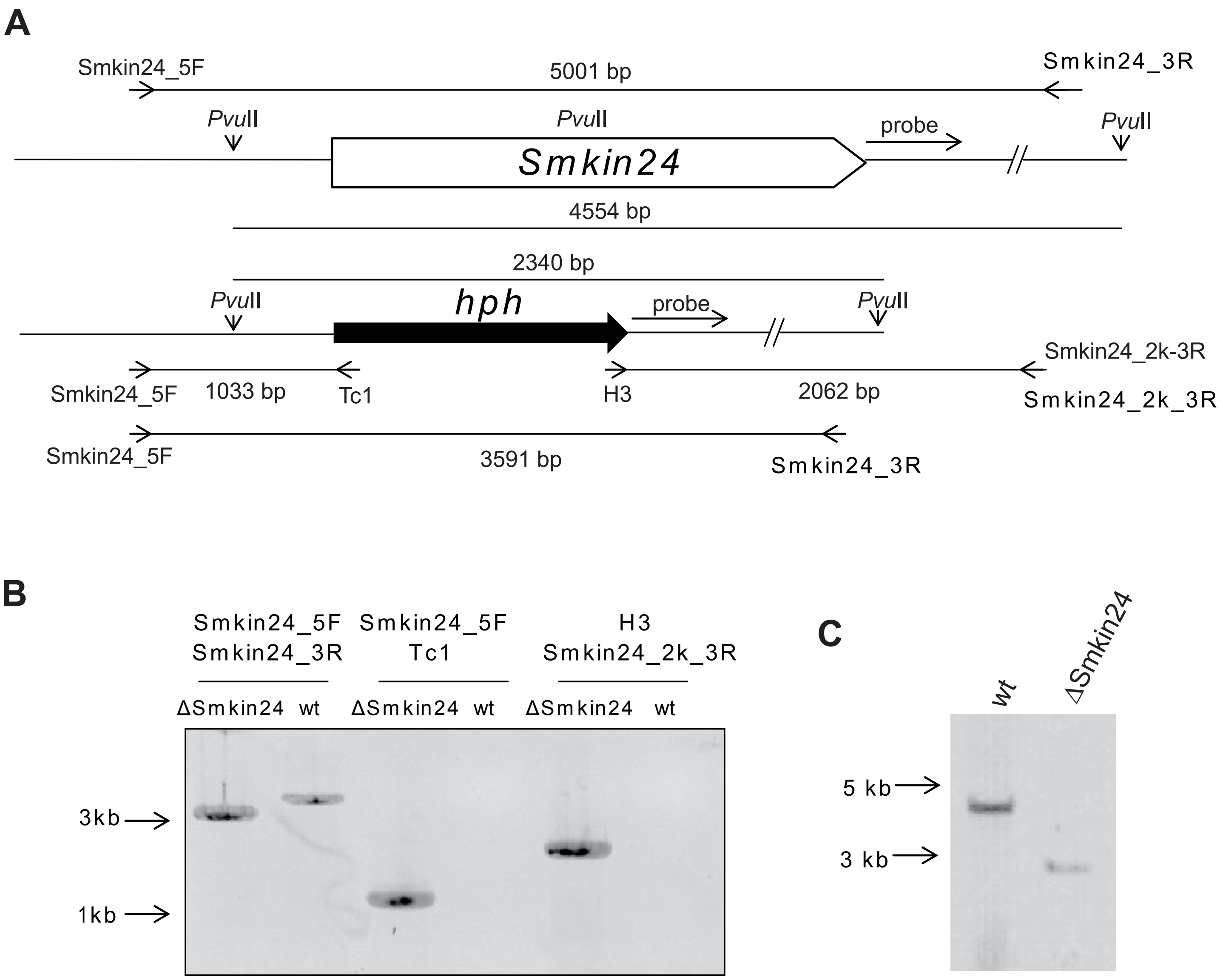
A
Smkin24_5F
5001 bp
Smkin24_3R
PvuII
PvuII
probe
PvuII
Smkin24
4554 bp
2340 bp
PvuII
hph
probe
PvuII
Smkin24_2k-3R
Smkin24_5F
1033 bp
Tc1
H3
2062 bp
Smkin24_2k_3R
Smkin24_5F
3591 bp
Smkin24_3R
B
Smkin24_5F
Smkin24_3R
Smkin24_5F
Tc1
H3
Smkin24_2k_3R
ΔSmkin24
wt
ΔSmkin24
wt
ΔSmkin24
wt
3kb
1kb
C
wt
ΔSmkin24
5 kb
3 kb

S1, Fig. G

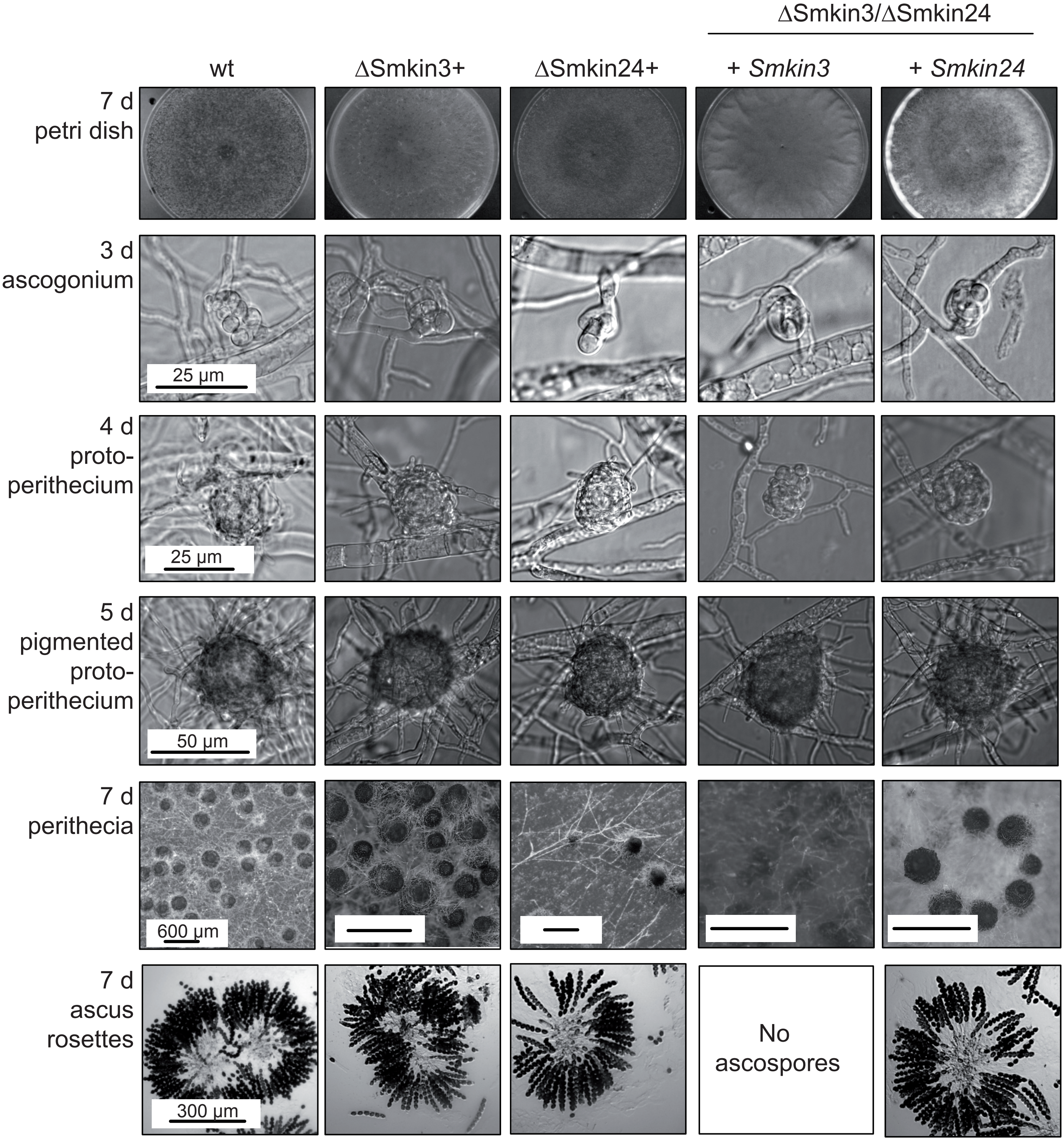
ΔSmkin3/ΔSmkin24
wt
ΔSmkin3+
ΔSmkin24+
+ Smkin3
+ Smkin24
7 d
petri dish
3 d
ascogonium
25 µm
4 d
proto-
perithecium
25 µm
5 d
pigmented
proto-
perithecium
50 µm
7 d
perithecia
600 µm
7 d
ascus
rosettes
300 µm
No
ascospores

S1, Fig. H

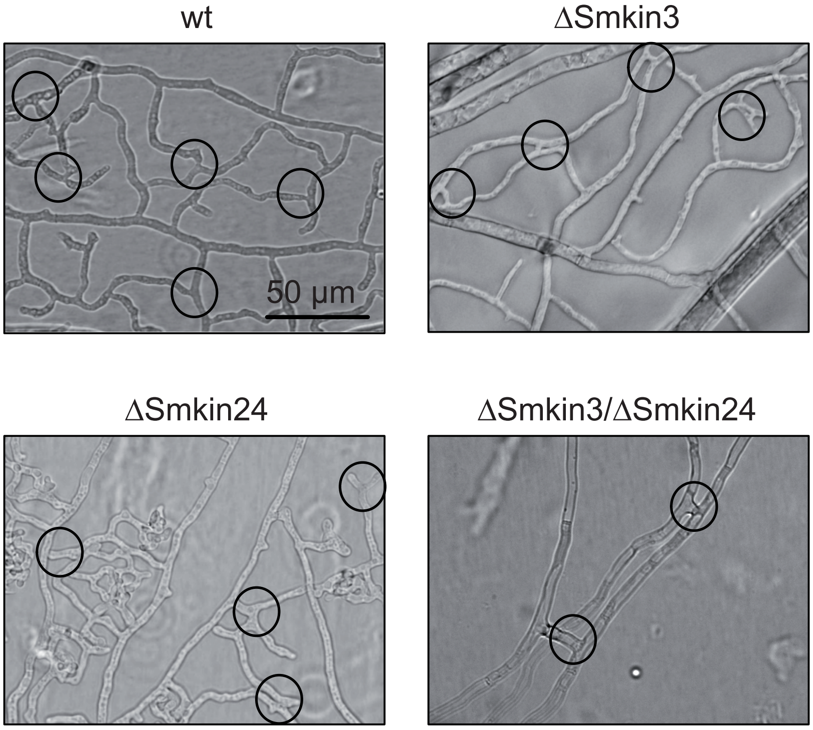

S1, Fig. I
